# Probabilistic Forecasting for Coarse-Grained Molecular Dynamics

**DOI:** 10.1021/acs.jctc.5c02131

**Published:** 2026-04-21

**Authors:** Luc F. Christians, Anna Wojnar, Alexander J. Pak

**Affiliations:** † Department of Chemical and Biological Engineering, 3557 Colorado School of Mines, Golden, Colorado 80401, United States; ‡ Quantitative Biosciences and Engineering Program, Colorado School of Mines, Golden, Colorado 80401, United States; § Materials Science Program, Colorado School of Mines, Golden, Colorado 80401, United States

## Abstract

Coarse-grained molecular dynamics enables access to long length and time scales but often fails to reproduce atomistic kinetics when memory effects and slow collective motions are important. We introduce Probabilistic Forecasting for Coarse-Graining (PFCG), a machine learning framework that learns stochastic coarse-grained equations of motion directly from atomistic trajectories by formulating coarse-grained simulation as a probabilistic time-series forecasting problem with both Markovian and non-Markovian contributions. PFCG incorporates non-Markovian effects through finite trajectory history without requiring explicit memory kernels or learned effective potentials. We apply PFCG to miniproteins and polyalanine peptides and evaluate both configurational and dynamical fidelity using free energy surfaces, autocorrelation functions, and transition time scales from Markov state models. Across all systems, non-Markovian PFCG models significantly improve dynamical agreement with atomistic simulation relative to Markovian baselines while also maintaining excellent agreement with stationary distributions. Additional tests of transferability show that PFCG remains robust under sparse sampling with few transition events, but in its current formulation does not extrapolate to metastable states absent from the training set. These results highlight the importance of inductive biases at the level of equations of motion and establish PFCG as a complementary approach to existing machine learning-based coarse-graining methods for modeling biomolecular processes.

## Graphical abstract

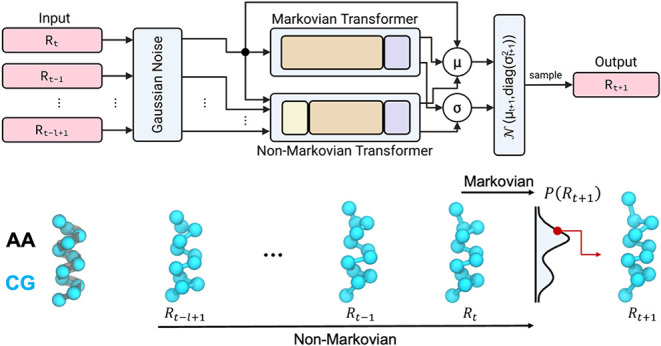

## Introduction

Classical molecular dynamics (MD) simulations serve as a computational microscope that enables the exploration of dynamical pathways and interactions within molecular systems at atomistic resolution, e.g., across biological, condensed matter, or soft matter systems.^1^ However, the length and time scales relevant to the behaviors of interest can be orders of magnitude larger than those accessible to atomistic MD simulations.^2^ Here, we focus on biomolecular systems, such as those involving proteins, lipids, nucleic acids, or carbohydrates, that involve a wide range of phenomena. Examples include slow conformational transitions on the order of μs to ms to collective spatial organization into higher-order complexes on the order of s to hr, each with associated length scales on the order of nm to μm, respectively.^3^ For these systems, the biomolecules of interest are solvated, typically hydrated with physiological concentrations of salt, with solvent atoms explicitly represented by particles. As a result, the primary computational limitation of MD simulations is associated with the increasing amount of solvent as both length and time scales increase.

Coarse-graining is the practice of representing sets of fine-grained (FG) particles (e.g., atoms) as pseudoparticles known as coarse-grained (CG) sites, thereby reducing the computational burden of MD simulations and facilitating studies at extended length and time scales.^4^ The CG mapping determines the computational savings of the CG model with larger FG-to-CG ratios commensurate with greater savings but usually at the expense of accuracy. Explicit solvent models, such as the Martini CG model, map the biomolecule (or solute) of interest while also mapping solvent molecules separately.^5^ Implicit solvent models, on the other hand, map the biomolecule (or solute) of interest but completely integrate out all solvent degrees of freedom.^6^ As a result, the latter models dramatically reduce the computational cost of CG MD simulations compared to the former and enable access to biologically relevant length and time scales.

One of the main challenges with implicit solvent CG models is that of accuracy, especially when coupled with the need for increasingly coarser (i.e., lower resolution) mappings. Motivated by the success of atomistic force-fields, as well as the ease of implementation, many CG models approximate effective CG interactions as pairwise-distance decomposed functions.^7–10^ In so-called bottom-up CG models, these pairwise interactions are systematically derived to match microscopic correlations that are sampled from FG statistics, often from all-atom (AA) MD simulations, with the goal to approximate the many-body potential of mean force (PMF) to enforce thermodynamic consistency.^9–13^ While demonstrably successful for simple organic fluids, polymers, and some globular proteins,^14–18^ the pairwise approximation is inadequate for systems with greater conformational disorder.

One natural way to improve accuracy is to incorporate higher-order interactions. For example, explicit three-body potentials^19,20^ or pseudothree-body potentials using virtual sites^21,22^ have been previously proposed. Data-driven approximations to generalized N-body interactions have also been demonstrated using machine learning (ML) approaches,^23–27^ similar to how machine-learned interatomic potentials (MLIP) coarse-grain electronic degrees of freedom into those that are classical.^14,28^ In CGNet, N-body interactions are defined based on combinations of CG pairwise distances input into parallel multilayer perceptrons (MLPs), each representing interactions that are two-body, three-body, and so on.^26^ Both DeePCG and CGSchNet learn effective CG interactions from the positions of neighboring CG sites, with the former leveraging a local frame transformation (LFT) and the latter using message-passing graph neural networks.^25,27^ All of these examples have been shown to recapitulate lower-dimensional projections of the many-body PMF, yet it is unclear if the FG dynamics are preserved by the CG model. One might expect that the removal of solvent also removes solvent damping effects, which are often approximated using friction terms constrained by the second fluctuation–dissipation theorem (FDT) such as in the Langevin equation^29^ (when integration is treated as a Markovian process) or the Generalized Langevin equation (GLE) when non-Markovian.^30,31^

We propose that an alternative approach to CG models that preserve both the fidelity of the many-body PMF and dynamics may be to learn data-driven approximations of the effective CG equations of motion directly. In the case of simple atomic systems, Kadupitiya and coworkers have shown that long short-term memory (LSTM) networks, a type of recurrent neural network, replicate trajectories given a short fragment of trajectory history while facilitating up to a 4000-fold increase in time step.^32^ Similarly, Tsai and coworkers have shown that LSTMs preserve the underlying dynamics of molecular systems, including alanine dipeptide, when trained on trajectories of low-dimensional collective variables that are tokenized following natural language processing practices.^33^ Ferguson and coworkers^34^ and Koumoutsakos and coworkers^35^ investigated the use of mixture density networks and variational auto encoders (VAE) to propagate reduced dimensionality latent representations of AA configurations. In all of these cases, the central principle is to forecast the next step of a trajectory given finite-length and discretized information on the past trajectory.

In this work, we investigate machine learning models specific for sequential data to forecast CG trajectories while addressing three specific challenges: (i) the inherent FG configurational degeneracy of CG sites, (ii) the non-Markovianity of the CG equations of motion primarily due to lost solvent degrees of freedom, and (iii) propagation of integration error when using the forecaster in an autoregressive manner. We call our approach Probabilistic Forecasting for Coarse-Graining (PFCG) and demonstrate its efficacy for two miniproteins (chignolin and Trp-cage) and two α-helical polyalanine peptides (A_5_ and A_12_). Our findings suggest that rather than separately learning effective CG forces and memory kernels (e.g., for GLE), one can indirectly learn the complete CG equations of motion using machine learning forecasting models.

## Methods and Theory

### Theory

We first define a CG trajectory that is discretized across time. The CG configurational variables are denoted as *R_t_* = {*r*_1,*t*_,*r*_2,*t*_,*r*_3,*t*_,...,*r_N_*,_*t*_} where *t* is an index over discretized time, *r*_*i*,*t*_ = (*x*_*i*,*t*_,*y*_*i*,*t*_,*z*_*i*,*t*_) refers to the coordinates of CG site *i* at time index *t*, and *N* is the number of CG sites. If the total time of the trajectory is *τ*, then *τ* = *N_t_* Δ*t* where Δ*t* is the time step, *N_t_* is the number of timesteps, and *t* = 0,1,2,...,*N_t_*. Note that *R_t_* can either be sampled during a CG MD simulation or mapped from an AA MD simulation using the mapping operator $M\left(R_{t}^{AA}\right)=R_{t}$ where $R_{t}^{AA}$ refers to the AA coordinates of the biomolecules (or solutes) at time index *t*.

When the time evolution of *R_t_* is treated as a Markovian process, it is often convenient to define the equations of motion for each CG site following the Langevin equation, a type of stochastic differential equation:

1 $$m_{i}\;\overset{¨}{r_{i,t}}=-\nabla_{r_{i}}U\left(R_{t}\right)-\gamma \overset{˙}{r_{i,t}}+\sigma \eta_{i}$$

where *m_i_* is the mass of CG site *i*, *U*(*R_t_*) is the potential energy of configuration *R_t_*, *γ* is the friction imposed by the environment (i.e., the implicit solvent), *η_i_* is a 3D vector of random numbers sampled from a normal distribution with zero mean and unit variance, and *σ* is the magnitude of the random force determined by the second FDT. This equation of motion assumes that there is a clear separation in slow solute and fast solvent degrees of freedom such that the decorrelation time of the latter is much faster than the former. When this Markovian approximation is not valid, one might instead use the GLE:

2 $$m_{i}\overset{¨}{r_{i,t}}=-\nabla_{r_{i}}U\left(R_{t}\right)-\int_{0}^{t\Delta t}K\left(t\Delta t-\mathrm{t̂}\right)\overset{˙}{r_{i,\mathrm{t̂}}}d\mathrm{t̂}+\sigma_{t}\eta_{i}$$

where *K* is a memory kernel that describes how the history of (solute-only) velocity degrees of freedom affects acceleration and the noise term *σ_t_* is now colored noise (i.e., time correlated) rather than white noise. While deriving an explicit form for *K* is quite challenging, approximations for *K* operating at CG resolution that are learned from AA statistics have been proposed.^31^ However, extending these solutions to CG dynamics driven by many-body interactions remains computationally and theoretically difficult.

A non-Markovian process can be reformulated into one that is Markovian by introducing auxiliary variables,^36,37^ often referred to as hidden variables *H_t_* = {*h*_1,*t*_,*h*_2,*t*_,...,*h_d_*,*_t_*} with dimensionality *d*. The so-called extended system of (*R_t_*,*H_t_*) can follow equations of motion similar to that of eq 1, which when numerically integrated take the form of (*R_t_*_+1_,*H_t_*_+1_) = *f*(*R_t_*,*H_t_*) where *f* is an unknown integrator from discretization of the underlying equation of motion for the extended system. Here, we assume that *H_t_* can be represented as a function of a finite history (or trajectory) of prior *R* values, i.e., *H_t_* = *g*(*R_t_*,*R*_t–1_,*R_t_*_–2_,...,*R_t_*_–1+1_) where *l* is the length of the history, similar to the memory kernel integral in eq 2. The inclusion of history in this manner can be thought of as an application of Takens’ delay embedding theorem, where the dynamics of a system can be learned even with “incomplete” knowledge of the system state (here, only knowledge of solute positions) using time-lagged examples.^38,39^ Since *H_t_* only depends on prior values of *R* up to *R_t_* (and likewise, *H_t_*_+1_ depends on prior values of *R* up to *R_t_*_+1_), the extended system integrator takes the simplified form:

3 $$R_{t+1}=f\left(R_{t},H_{t}\right)=f\left(R_{t},g\left(R_{t},R_{t-1},R_{t-2},...,R_{t-l+1}\right)\right)$$

where the impact of the auxiliary variables is now embedded in the functions *f* and *g*.

The integration model suggested by eq 3 addresses the possible non-Markovian nature of CG solute (or biomolecule) dynamics in solvent, but we must consider one more aspect. Both eqs 1 and 2 contain a stochastic term that captures the impact of configurational variability of absent solvent molecules. However, we should also consider the fact that the CG mapping operator introduces a mapping entropy where CG configuration *R_t_* is degenerate to multiple $R_{t}^{AA}$ configurations. In other words, a given *R_t_* can evolve into a distribution of possible *R*_*t*+1_ even if all AA degrees of freedom are explicitly mapped (e.g., none of the solvent degrees of freedom are integrated out). We capture both of these sources of stochasticity into a unified representation:

4 $$P\left(R_{t+1}|R_{t},...,R_{t-l+1}\right)=f\left(R_{t},H_{t}\right)=f\left(R_{t},g\left(R_{t},R_{t-1},R_{t-2},...,R_{t-l+1}\right)\right)$$

where *P*(*R_t_*_+1_|*R_t_*,...,*R*_*t*–*l*+1_) refers to the conditional probability distribution for configuration *R_t_*_+1_ given a prior trajectory of *R* with length *l*. Training CG integrators that recapitulate eq 4 is the central idea of this work. One can interpret *f* as the function that computes the probability distribution with *g* the function that computes the set of hidden variables. Here, for simplicity, we assume that *R*_*t*+1_ is sampled from a multivariate normal distribution with a diagonal covariance matrix, i.e.:

5 $$R_{t+1}∼Q\left(R_{t+1}|R_{t},...,R_{t-l+1}\right)=N\left(\mu_{t+1},\mathrm{diag}\left(\sigma_{t+1}^{2}\right)\right)$$

where $\mu_{t+1}\in R^{3N}$ and $\sigma_{t+1}\in R^{3N}$ are the vectors of mean and standard deviations, respectively, for each CG degree of freedom in *R_t_*_+1_ and *Q*(*R_t_*_+1_|*R_t_*,...,*R_t_*_–*l*+1_) is our approximation to *P*(*R_t_*_+1_|*R_t_*,...,*R_t_*_–*l*+1_). Inspired by eq 2, we split the contributions to *μ_t_*_+1_ and *σ*_*t*+1_ such that

6 $$\mu_{t+1}=R_{t}+\mu_{m}+\mu_{nm}$$

7 $$\sigma_{t+1}=\sigma_{m}+\sigma_{nm}$$

8 $$\left[\mu_{m},\;\sigma_{m}\right]=F_{m}\left(R_{t};\;\theta_{m}\right)$$

9 $$\left[\mu_{nm},\sigma_{nm}\right]=F_{nm}\left(R_{t},R_{t‐1},...,R_{t‐l+1};\;\theta_{nm}\right)$$

where $F_{m}$ and $F_{nm}$ are neural network models with respective trainable parameters *θ_n_* and *θ_nm_*, each representing the Markovian (i.e., the effective force) and non-Markovian (i.e., the effective memory kernel) contributions to eq 2. In the following sections, we describe our architecture for $F_{m}$ and *F_nm_*, as well as our training strategy to ensure that *Q*(*R_t_*_+1_|*R_t_*,...,*R_t_*_–l+1_) variationally approximates *P*(*R_t_*_+1_|*R_t_*,...,*R_t_*_–*l*+1_).

### Model Architecture

As shown in Figure 1, we construct the PFCG model in two forms (Markovian and non-Markovian) from four base components: (1) the Gaussian noise component that adds noise to input data (only during training), (2) a Markovian component that is learned from many-body configurational correlations, (3) a non-Markovian component that is learned from “many-body” correlations across hidden state variables, which in turn are learned from the provided history of configurational variables, and (4) the output layer that combines the learned parameters describing the probability distribution of the next step of the trajectory. The non-Markovian model employs all four components while the Markovian model skips the third component. Hyperparameters for the described components are summarized in Table S5.

**Figure 1:**
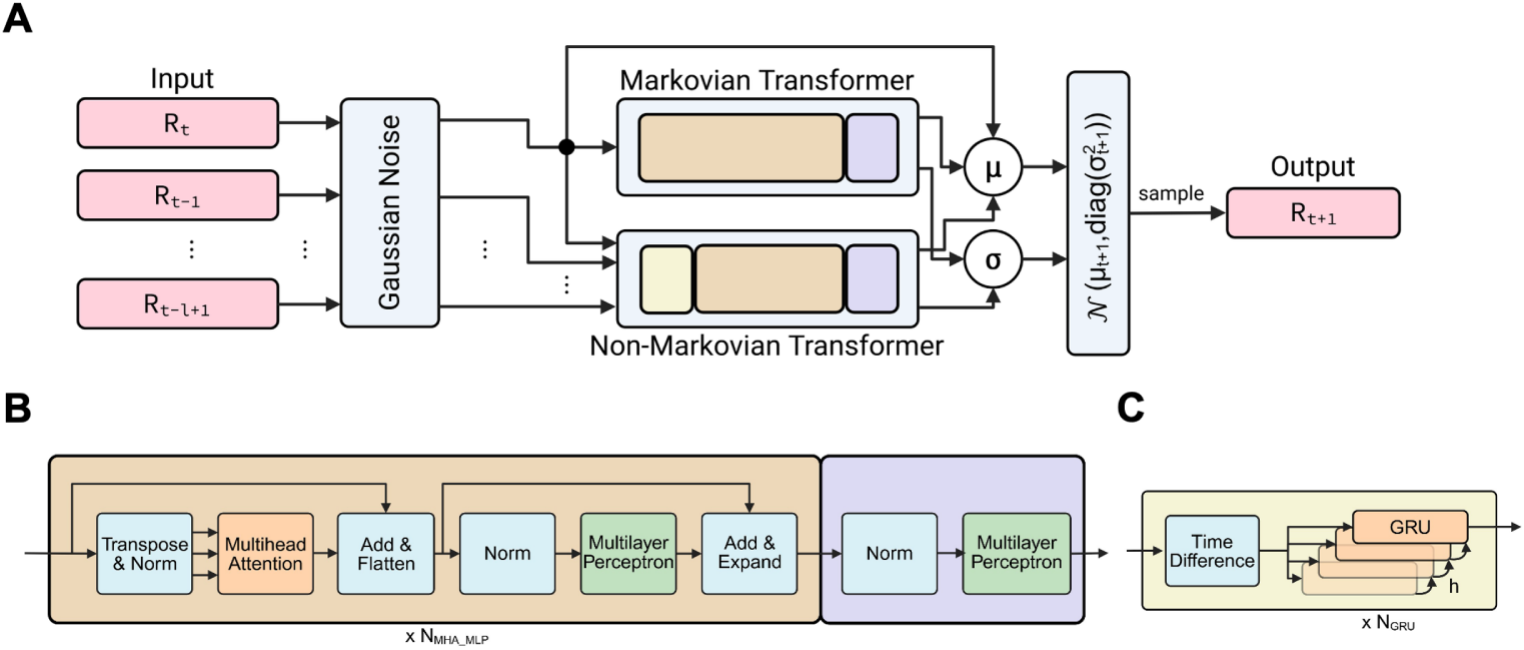
The neural network architectures for PFCG. (A) Schematic of the overall architecture. The history of input configurations (pink) is first conditioned with random Gaussian noise. Next, the data is fed into the Markovian and non-Markovian layers, with the Markovian layer receiving only the single, most recent configuration *R_t_*. Note that *R_t_* is split three ways and sent to both the Markovian and non-Markovian layers, as well as the final *μ* addition operation. Both layers output additive contributions to the predicted mean (*μ_t_*_+1_) and standard deviation (*σ*_*t*+1_) of the output probability distribution $N\left(\mu_{t+1},\mathrm{diag}\left(\sigma_{t+1}^{2}\right)\right)$ . The next configuration *R*_*t*+1_ is sampled from the resultant probability distribution. (B) The portion of the Transformer block that is shared by both the Markovian and non-Markovian layers. The brown block shows the attention mechanism, which is repeated *N_MHA_*___ *_MLP_* times. The purple block shows the final multilayer perceptron layer that predicts *μ* and *σ*. (C) The initial portion of the non-Markovian Transformer block (yellow block) first computes a time-differenced sequence of the history of input configurations. The data is then processed through gated recurrent units (GRUs) with *N_GRU_* layers to predict the sequence of hidden auxiliary variables (*h*), which are sequential passed as input to the next GRU node. The *h* values from the final GRU node are the output. Created in BioRender. Christians, L. (2026) https://BioRender.com/6l013c0.

The Gaussian noise component, which is turned off during inference (i.e., CG MD integration), is included to help the model learn generalized behavior during MD integration by seeing small perturbations to the training data. The added noise is centered at zero with a standard deviation *σ*_*g*_ that we set as a hyperparameter. Accumulation of error during inference is a common problem with recurrent neural networks and other forecasting model architectures.^40^ We had tried a few mitigation strategies, such as scheduled sampling and professor forcing,^41,42^ but found that adding Gaussian noise to our input data (trained in teacher forcing mode) achieved comparable results with faster training times and more robust convergence.

The Markovian component takes as input the most recent configuration *R_t_* from the Gaussian noise component and learns [*μ_m_*,*σ_m_*] via an MLP informed by many-body spatial correlations that are identified using a transformer-inspired architecture^43^ (see Figure 1B). The input data has dimensions of *N_b_* × 1 × 3*N* where *N_b_* is the batch size and 3*N* is the number of LFT features. We transpose the last two dimensions such that self-attention via the Multihead Attention layer is learned on the features themselves. After the residual connection to the input data (prior to normalization), we flatten the last two dimensions and perform a nonlinear transformation via an MLP with two hidden layers. We then perform another residual connection and expand the dimensionality back to *N_b_* × 1 × 3*N*. This process is repeated for *N_MHA_*___ *_MLP_* cycles. The output then enters a final MLP with two hidden layers (here, random dropout is applied during training). The output layer is linear (without activation) and doubles the dimensionality to *N_b_* × 1 × 6*N*, with the first 3*N* elements representing *μ_m_* for each feature and the remaining 3*N* elements representing *σ_m_* for each feature. Throughout, we apply layer normalization to stabilize training.^44^

The non-Markovian component is similar to the Markovian component but instead takes as input the sequence {*R_t_*,...,*R_t_*_–*l*+1_} (with dimensionality *N_b_* × *l* × 3*N*) from the Gaussian noise component and learns [*μ_nm_*,*σ_nm_*] informed by many-body correlations in *H_t_*. To compute *H_t_*, we first time-difference the input data as *R_j_*–R_*j*–1_ (yielding dimensionality *N_b_* × *l*–1 × 3*N*) as a proxy for velocities, then encode *H_t_* from two gated recurrent unit (GRU) layers^45^ (see Figure 1C) yielding dimensionality *N_b_* × 1 × *h_dim_* where *h_dim_* is the size of *H_t_*. Then, *H_t_* proceeds through the same transformer-inspired model (see Figure 1B) and yields a final prediction of size *N_b_* × 1 × 6*N* representing the *μ_nm_* and *σ_nm_* for each feature. Here, the GRU layer serves as a short-term integrator for *H_t_* and we facilitate temporal consistency by simultaneously training five one-step forward predictions within each batch, i.e., we predict the output for *R*_*t*+1_ through *R*_*t*+5_ simultaneously (but only during training).

The final output of the PFCG model is the set of *μ*_*t*+1_ and *σ*_*t*+1_ values representing the output probability density for *R*_*t*+1_as described by eqs 5–9. For Markovian models, the final *μ* is the sum between *R_t_* and that of the Markovian component (*μ_m_*) while the final *σ* is just that of the Markovian component (*σ_m_*). For non-Markovian models, the *μ_nm_* and *σ_nm_* from the non-Markovian component is included as shown in eqs 6–7. In all cases, the final *μ*_*t*+1_ is activated with a hyperbolic tangent function then linearly scaled to −0.75 to 0.75, the expected range for features.

Taking the totality of the PFCG model architecture, the final output is *Q*(*R_t_*_+1_|*R_t_*,...,*R_t_*_–*l*+1_). The model is trained by minimizing the negative log likelihood loss function L :

10 $$L=‐\frac{1}{N_{b}}\frac{1}{3N}\overset{N_{b}}{\underset{s=1}{\sum }}\overset{N}{\underset{i=1}{\sum }}\overset{3}{\underset{d}{\sum }}\ln\left(Q\left(r_{i,t+1,d}^{s}|R_{t}^{s},...,R_{t‐l+1}^{s}\right)\right)$$

where the index *s* is over all samples within the batch and $r_{i,t+1,d}^{s}$ is the true LFT position for site *i*, dimension *d* (for *x*, *y*, or *z*), time *t*+1, and sample *s*. As previously discussed by Tsai and co-workers in the context of tokenized probability vectors but without loss of generality,^33^ minimization of L is equivalent to minimization of the cross-entropy *J* between $P\left(R_{t+1}|R_{t},...,R_{t-l+1}\right)≡P_{t+1,l}$ , the true conditional probability over paths of length *l*, and the learned conditional probability $Q\left(R_{t+1}|R_{t},...,R_{t-l+1}\right)≡Q_{t+1,l}$ , with *J* rewritten as

11 $$J=\frac{1}{N_{b}}\frac{1}{3N}\overset{N_{b}}{\underset{s=1}{\sum }}\overset{N}{\underset{i=1}{\sum }}\overset{3}{\underset{d}{\sum }}\left[-P_{t+1,l}\ln\left(P_{t+1,l}\right)+P_{t+1,l}\ln\left(\frac{P_{t+1,l}}{Q_{t+1,l}}\right)\right]$$

where the second term on the left-hand side is the Kullback-Liebler divergence between $P_{t+1,l}$ and $Q_{t+1,l}$ and the first term is the path entropy.^33^ Since the global minimum of *J* (and L ) is obtained when $P_{t+1,l}=Q_{t+1,l}$ , minimization of L effectively leads to learning the path entropy. In the following sections, we will discuss how training data is obtained, how training is performed, and how models are assessed in terms of both stationary and dynamical properties.

### Atomistic Molecular Dynamics Simulations

Atomic structures for chignolin (PDB 1UAO^46^) and Trp-cage (PDB 1L2Y^47^) were obtained from the Protein Data Bank, and polyalanine structures were generated in an alpha helical state using peptideBuilder^48^ (Figure 2A). Atomistic data sets were generated for all proteins using the CHARMM36m force field^49^ for proteins and the modified TIP3P force field for water^50^ using GROMACS (version 2023.1).^51^ All proteins were explicitly solvated using TIP3P water in a 0.15 M NaCl aqueous solution with parameters defined in Tables S1–2. Geometry optimization was performed using steepest descent to a threshold of 500 kJ/mol/nm. All systems were equilibrated to their target temperatures using the stochastic velocity rescaling thermostat^52^ in the constant NVT ensemble for 5.0 ns, then equilibrated to 1.0 bar using the Parrinello–Rahman barostat^53^ in the constant NPT ensemble for 10 ns. A final equilibration in the constant NVT ensemble was performed for a total time of *τ_e_*. After equilibration, long production simulations were conducted in the constant NVT ensemble for a total time of *τ_L_* saved every Δ*t_L_*, as we then subsampled trajectories with data saved at a high frequency from the long production trajectories (see Figure 2B). All simulations used a 2.0 fs time step with LINCS constraints on hydrogen-containing bonds. All simulation parameters are summarized in Table S3. To create high-frequency trajectories used for model training, we extracted *N_D_* initial configurations and velocities from the long production trajectories; initial states were chosen to be equally spaced along the long production trajectories. Simulations were run under identical conditions to the production simulations except all trajectories were saved at a frequency of Δ*t_D_* = 50 fs over *τ_D_* per trajectory. These simulations were run long enough to ensure complete configurational sampling by comparing the aggregate dense production data to the long production data in the form of the similarity between free energy surfaces (FESs). In total, we collected 2.0 μs, 1.5 μs, and 5.0 μs of data for our chignolin, Trp-cage, and polyalanine systems (shown in Figure 2A), respectively.

**Figure 2:**
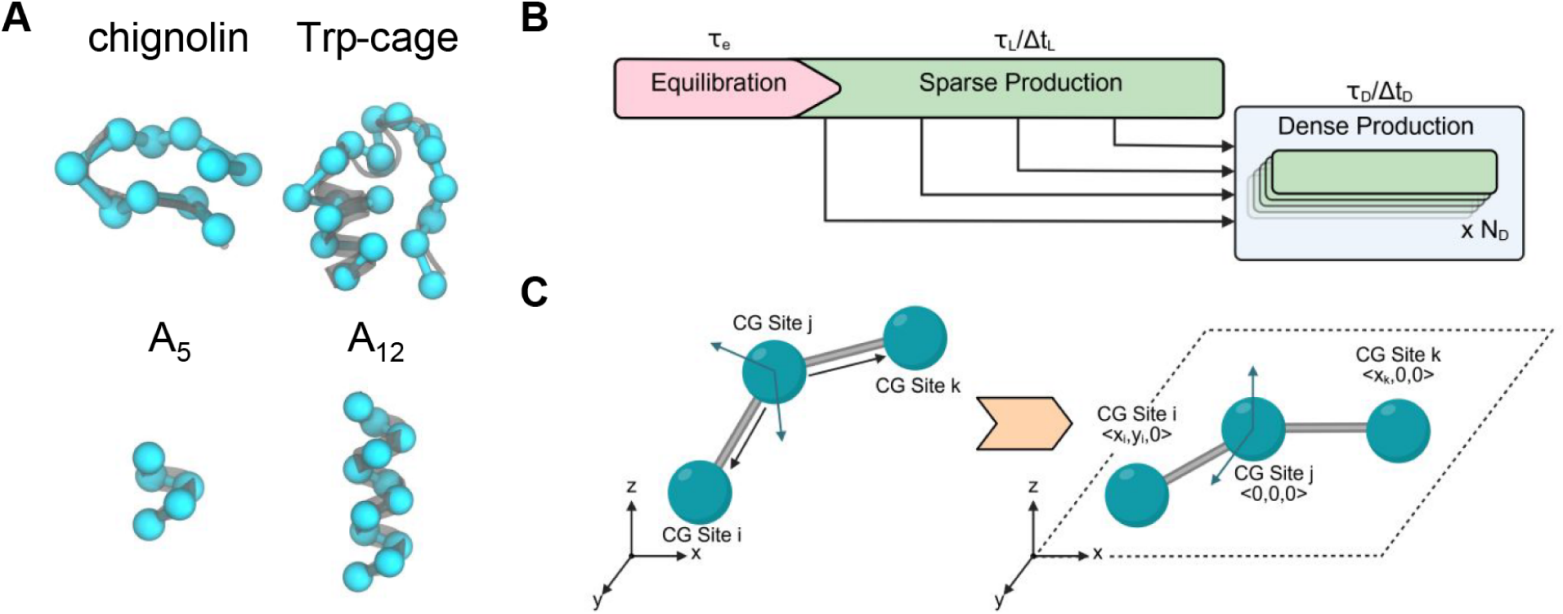
Data preparation and test systems. (A) Depiction of mini-proteins (chignolin, Trp-cage, A_5_, and A_12_) investigated in this study. The transparent gray surface is the atomistic model in New Cartoon representation while the cyan beads represent the mapped CG sites. (B) Schematic of workflow to generate the atomistic data sets. After equilibration, a long production simulation was run with infrequent data saving. Saved points were used to launch sets of short simulations with data saved at high-frequency. (C) Schematic of the local frame transformation featurization strategy, where the molecule is translated such that reference atom *j* is at the origin, then rotated such that reference atoms *i-j-k* are aligned to the *xy* plane. Partially created in BioRender. Christians, L. (2026) https://BioRender.com/a0zlpg4 and https://BioRender.com/c1a97q7.

### Model Training and Inference

The short, high-frequency AA MD trajectories were processed for CG model training and analysis. Each protein was CG mapped to a single CG site per residue, using the center of mass of each residue. We then applied the LFT to each frame via translation and rotation dictated by three reference CG sites *i, j,* and *k* in each protein (see Table S4 and Figure 2C). After translating the entire protein such that CG site *j* is at the origin, we applied the following transformation:

12 $$\mathrm{F̂}=Q_{rot}X=\left[\begin{array}{l} \overset{⃗}{r_{jk}\;}\\ r_{jk}\times \left(\overset{⃗}{r_{jk}\;}\times \overset{⃗}{r_{ji}\;}\right)\\ \left(\overset{⃗}{r_{jk}\;}\times \overset{⃗}{r_{ji}\;}\right) \end{array}\right]\left[\begin{array}{lll} x_{1} & ... & x_{n}\\ y_{1} & ... & y_{n}\\ z_{1} & ... & z_{n} \end{array}\right]$$

where F̂ is the matrix of transformed CG site coordinates, *Q_rot_* is the rotation matrix defined by the vectors $\overset{⃗}{r_{jk}\;}$ and $\overset{⃗}{r_{ji}\;}$ , and *X* is the matrix of Cartesian CG site coordinates after translation of CG site *j* to the origin. Our transformation ensures that our unscaled LFT features F̂ are translationally and rotationally invariant in an approach similar to but not the same as the one used by DeePCG.^27^ As a final step, we standardized F̂ using the MinMaxScaler from *scikit-learn* into the range −0.75 to 0.75, which we refer to as scaled LFT features *F*. Note that six degrees of freedom are always zero upon this transformation, and we enforce this condition during training and inference. We next partitioned the trajectories into data sets dependent on (CG integration time step) Δ*t* and (input history length) *l*. We extracted windows of length *l*+1 with Δ*t* spacing between frames with the first *l* frames for the input sequence of the model and the last frame as the true output prediction of the model. Data sets were downsampled further to yield around a million training samples per protein to mitigate memory usage and training time; we confirmed that the downsampled data set maintains ensemble statistics (via FESs) compared to the full data set. Each data set was then randomly shuffled and split into 70% training, 15% validation, and 15% test data.

Hyperparameters for model architectures were optimized through hand tuning, as we found that negative log likelihood losses below around −2.0 were not indicative of model performance during inference (Figure S1). To begin, Markovian models were optimized for each system at each desired Δ*t* based on the lowest Jensen-Shannon divergence (described later). In this process, models were trained using the Lion optimizer^43,54^ using a learning rate of 5 × 10^–5^ and a weight decay factor of 0.1 with early stopping triggered after seven epochs. In all cases, *N_b_* was set to 256 and *σ_g_* was set to 0.01. The lowest validation loss models were saved and used to generate a test loss from the test data set.

After model training, we performed CG MD simulations with initial configurations (and input histories) from 128 randomly selected windows from the test data set. All 128 replicas were run in tandem to take advantage of tensor operations. Simulations were integrated for 200 ns with data saved every 10 ps. After each integration time step, a configurational sample was generated from the learned PFCG probability distribution, representing the *R*_*t*+1_ configuration. These results were appended to the PFCG input history (with the oldest configuration subsequently removed) and the process was repeated until completion.

The stationary probability distributions were projected onto 2D time-lagged independent component analysis (TICA) space, with the TICA model trained using the unscaled LFT features F̂ from the AA MD simulations using a lag time of 500 ps. To ensure comparability, the generated PFCG trajectories of F̂ were unscaled back to F̂ prior to their projection in TICA space. The PFCG distributions were compared to that of AA MD using the Jensen-Shannon divergence:

13 $$D_{JS}\left(P_{AA}\left|\right|P_{CG}\right)=\frac{1}{2}\sum P_{AA}\ln\left(\frac{P_{AA}}{\frac{1}{2}\left(P_{AA}+P_{CG}\right)}\right)+\frac{1}{2}\sum P_{CG}\ln\left(\frac{P_{CG}}{\frac{1}{2}\left(P_{AA}+P_{CG}\right)}\right)$$

where *P_AA_* and *P_CG_* refer to the AA and CG 2D configurational probability mass functions, respectively. Note that *D_JS_* goes to zero if the two probability distributions are the same, with larger divergences indicating larger differences between the two distributions up to an upper bound of ln(2). The 2D potential of mean force (PMF) was computed from the 2D probability densities via −*k_B_T*ln­(*P*(*TIC*_1_,*TIC*_2_)), where *k_B_* is the Boltzmann constant and *T* is temperature; we shifted the PMFs such that the minimum value is at zero.

Once the Markovian model was trained, the next step was to train the non-Markovian component. During this stage, the weights of the Markovian component were fixed such that only the weights of the non-Markovian component were trainable. To simplify hyperparameter optimization, we applied the same hyperparameters from the Markovian component to the non-Markovian component. Instead, we focused hyperparameter tuning on the size of *H_t_*, where we considered multiples (2–4x) of the number of *F* features. We also tested different values of *l*, as discussed later.

To assess dynamical behavior, we computed the autocorrelation function (ACF) for each TICA component *i* of interest (TIC*_i_*) via:

14 $$ACF_{i}\left(\delta t\right)=\frac{<TIC_{i}\left(0\right)TIC_{i}\left(\delta t\right)>}{<TIC_{i}\left(0\right)TIC_{i}\left(0\right)>}$$

where *δt* is the lag time. The ACF was calculated up to *δt* = 5 ns for both the AA and CG MD simulations. The difference between the AA and CG ACFs was quantified using the mean squared error (*ϵ*_ACF_) where zero indicates perfect overlap.

In the case of polyalanine, we also trained Markov state models (MSMs) from the AA and CG trajectories as we expect polyalanine to have multiple intermediate states between the random coil and *α*-helical states.^55,56^ The MSMs were trained using TICA features with clustering performed in two steps: first, 250 K-Means clusters (with k-means++ initialization) were found, which were then coarsened into 2 macrostates using PCCA+ clustering.^57^ To select the lag times for MSM construction, we analyzed implied time scales, VAMP scores,^58^ and Chapman-Kolmogorov (CK) tests^59^ with results reported in Figures S2–5 for each AA model. All cluster definitions and TICA transformations were trained using the AA data set then applied to the CG data set. All MSMs were trained using the Deeptime 0.4.4 Python package.^60^ The mean free passage times (MFPT) between macrostates were assessed from the MSMs and used to quantity transition dynamics. The difference between the AA and CG MFPTs were assessed using mean absolute relative error (*MARE_MFPT_*).

## Results and Discussion

### Impact of Hyperparameters on Mini-Protein Molecular Dynamics

We evaluate the fidelity of the PFCG model for chignolin^46^ and Trp-cage,^47^ two mini-proteins that have been well-characterized in prior studies.^61–64^ We generated AA MD trajectories as described above and all simulation setup parameters can be found in Tables S1–S3. We note that we sampled chignolin and Trp-cage at 420 and 270 K, respectively, to accelerate and to suppress conformational sampling as two representative extrema of intended PFCG use cases. To create training data, each system was coarse-grained using center-of-mass mapping at a resolution of one CG site per residue, resulting in 10 CG sites for chignolin and 20 CG sites for Trp-cage. The CG positions were LFT featurized (i.e., 30 and 60 features total for chignolin and Trp-cage, respectively, with 6 features always at zero) and then prepared into sliding windows of varying input history lengths as described in Methods and Theory.

The primary hyperparameters of interest are the trajectory time step (Δ*t*) and the length of the input sequence (*l*), representing the history of the trajectory used to internally compute *H_t_* by the model. We tested varying Δ*t* of 1.0, 2.5, and 10.0 ps for both systems to see how robust the PFCG model is to large integration timesteps. By varying *l* (i.e., the length of the input sequence), we investigated the fidelity of the Markovian approximation (i.e., *l* = 1) compared to supplementing the Markovian model with increasing amounts of non-Markovian history (i.e., *l* >1). We first tuned the Markovian model for each system to determine optimal hyperparameters, as a low-fidelity Markovian model negatively impacted the performance of the corresponding non-Markovian model. The same hyperparameters of the Markovian transformer block were used in the non-Markovian transformer block. Details about complete model architecture, training, and trajectory generation hyperparameters can be found in Table S5.

We found model test losses decrease when supplementing the Markovian model with non-Markovian history, although test loss was not sensitive to increased *l* once non-Markovian history was added, as seen in Figure S6. We instead assessed model quality through analysis of the trajectories that were generated using the trained models (see Methods and Theory). We compared the 2D PMFs from AA and CG MD trajectories projected on the first two TICA components, TIC1 and TIC2, with TICA trained on unscaled LFT features from AA MD. We also computed *D_JS_* to assess the similarity between the AA and CG 2D configurational probability distributions of TIC1 and TIC2, where lower *D_JS_* up to a lower bound of zero indicates perfect overlap. Resultingly, lower *D_JS_* values also imply closer agreement between the AA and CG PMFs. To assess the quality of captured dynamics, we computed *ϵ_ACF_*, which represents the summed MSE loss between AA and CG ACFs for TIC1 and TIC2, with zero indicating a perfect match.

### Chignolin

Chignolin is a 10-residue mini-protein that folds into a β-hairpin structure.^46^ Chignolin has been the subject of both AA MD and CG MD studies due to its fast-folding behavior and small size.^25,61,62^ We originally trained PFCG models using Δ*t* = 0.05 ps (see Figure S7) and expectedly found that our Markovian model (*l* = 1) qualitatively reproduced the stationary distribution with accelerated dynamics compared to reference AA MD statistics. However, we found that the non-Markovian model (see Figure S7) trained using an input sequence length *l* = 75 (our maximum practical length due to memory limitations for chignolin and Trp-cage) yielded overly damped dynamics (i.e., slower than the reference AA MD statistics) and poor sampling of the stationary distribution. To adequately capture dynamics, we hypothesized that the history information should be long enough to capture the decorrelation of the time-differenced input features (i.e., our proxy for velocities). Indeed, we find that at Δ*t* = 0.05 ps, the averaged ACF profile across all time-differenced input features from the AA MD trajectories do not converge to zero within 100 lagged time points (see Figure S8).

To test longer history, we opted to increase Δ*t* to 1.0 ps and further tested longer timesteps of 2.5 and 10.0 ps. At *l* = 75, we are effectively including a history of length 75 ps at Δ*t* = 1.0 ps up to 750 ps at Δ*t* 10.0 ps. Unlike Δ*t* = 0.05 ps, the averaged ACF profiles across time-differenced features for all tested timesteps converge to zero on the same order of magnitude as *l* = 75 (Figure S8B). We note that the input trajectory lengths remain up to an order of magnitude shorter than the decorrelation times computed from AA MD for TIC1 (4.90 ns) and within the same order of magnitude as that of TIC2 (0.36 ns). The resulting *D_JS_* and *ϵ_ACF_* computed using PFCG models with Δ*t* = 1.0, 2.5, and 10.0 ps and increasing *l* can be found in Figure 3. The complete set of FESs and ACF curves for all tested *l* and Δ*t* can be found in Figures S9, S10, and S11.

**Figure 3:**
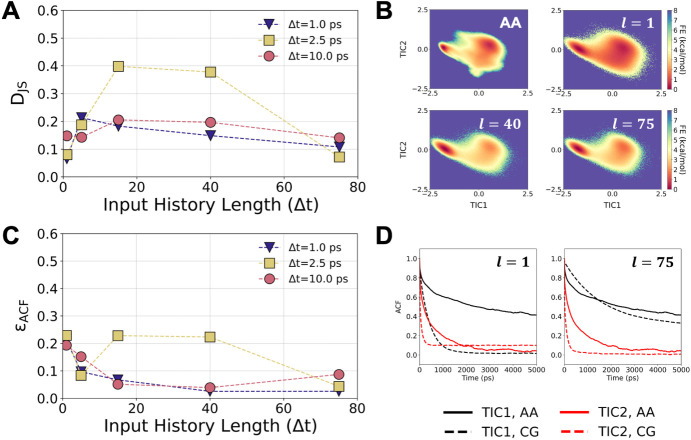
PFCG results across input history length and time step for chignolin. (A) Summary plot of the 2D Jensen-Shannon divergence (*D_JS_*) as a function of input history length (*l*), testing timesteps Δ*t* = 1.0 ps (triangle marker), 2.5 ps (square marker), and 10.0 ps (circle marker). (B) 2D free energy surfaces (FESs) across TIC1 and TIC2 from AA MD (top left) and from PFCG models at Δ*t* = 1.0 ps with *l* = 1 (top right), *l* = 40 (bottom left), and *l* = 75 (bottom right). (C) Summary plot of ACF error (*ϵ_ACF_*) as a function of input history length across tested timesteps. (D) Comparison of ACF curves for TIC1 and TIC2 calculated from AA MD and PFCG at Δ*t* = 1.0 ps with *l* = 1 (left) and *l* = 75 (right).

Regarding the ability of PFCG to recapitulate configurational statistics from AA MD for chignolin, we find in Figure 3A that increasing *l* has variable impact to *D_JS_*. At Δ*t* = 1.0 ps (triangle markers in Figure 3A), the Markovian model (*l* = 1) results in a low *D_JS_* of 0.066. Adding a short amount of history (*l* = 5) appears to increase *D_JS_* (to 0.213) with respect to the Markovian model, before a sufficient amount of history with *l* = 75 recovers a *D_JS_* of 0.107 that is comparable to that of the Markovian model. For both Δ*t* = 2.5 ps and Δ*t* = 10.0 ps, similar trends are observed where *D_JS_* initially increases with intermediate amounts of tested history lengths before *D_JS_* decreases at *l* = 75 and recovers distributions similar to that of the Markovian model. According to the FESs (Figure S10), the worsened performance at intermediate history lengths is due to undersampling of the unfolded state (and therefore, increased lifetime of the folded state), which suggests that an “incomplete” history for our effective dynamics leads to suppressed conformational fluctuations, similar to behavior we observed using Δ*t* = 0.05 ps (Figure S7).

Focusing on Δ*t* = 1.0 ps, we next investigate the impact of increasing *l* on the FESs, as shown in Figure 3B. All PFCG models capture the full phase space of the AAMD distribution, where the folded state is observed around (TIC1, TIC2) = (−1.8, 0.1) and the broader basin with a minimum around (0.55, 0.40) is the unfolded state. When *l* = 1, the PFCG trajectories overrepresent the unfolded state, which is commensurate with a lower energy barrier separating the folded and unfolded states relative to AA MD. As more history information is incorporated into training, the distributions more closely match that of AA MD. Both *l* = 40 and *l* = 75 capture the energy barrier between the condensed folded region and the broad unfolded region, with *l* = 75 better capturing the minimum in the unfolded region. Nonetheless, it is important to note that both the Markovian and non-Markovian models qualitatively capture the AA MD distribution, indicating that the “many-body” correlations inherent in the Markovian model are minimally sufficient to recapitulate the stationary distribution. This result is unsurprising since previously reported neural network-based CG force-fields also do the same.^27,65^

Next, we perform dynamical analysis as summarized in Figure 3C. We find that adding any amount of non-Markovian history results in a decrease in *ϵ_ACF_*, with larger *l* resulting in lower *ϵ_ACF_*. As lower *ϵ_ACF_* indicates closer agreement between the AA and PFCG ACF profiles for both TIC1 and TIC2, the inclusion of non-Markovian information improves the recapitulation of AA MD dynamics. Representative comparisons of ACF profiles for the *l* = 1 (Markovian) and *l* = 75 (non-Markovian) models are shown in Figure 3D. The ACF of the Markovian (*l* = 1) model shows much faster decorrelation compared to that of AA MD, which is especially apparent with TIC1 decorrelating within 2,000 ps. On the other hand, the non-Markovian (*l* = 75) ACF profile for TIC1, which corresponds to the slowest motion in chignolin, shows a decorrelation time scale comparable to that of AA MD. We note that the motion encoded by TIC2 still decorrelates too quickly for both the Markovian and non-Markovian models, suggesting that the current non-Markovian PFCG model primarily corrects the dynamics of the slowest mode.

### Trp-cage

Trp-cage is a 20-residue, fast-folding mini-protein that maintains its folded structure with maximal propensity around 277 K.^47,64^ Our 270 K AA MD simulations similarly do not undergo major configurational transitions, which is also reflected in our computed decorrelation time of 18.5 and 12.9 ns for TIC1 and TIC2, respectively (an order of magnitude slower than that of chignolin). Similarly to chignolin, we find at a Δ*t* = 0.05 ps, the PFCG model is unable to recapitulate atomistic dynamics at our maximum input history length of *l* = 75 (Figure S12). In Figure S13B, we show that the averaged ACF profile across all time-differenced features using Δ*t* = 0.05 ps converges to zero at a lag of 157 time-lagged points, which is still beyond *l* = 75. Therefore, we also assessed PFCG models for Trp-cage at Δ*t* = 1.0, 2.5, and 10.0 ps with input history lengths spanning *l* = 1 through *l* = 75; all results are summarized in Figure 4. For all tested timesteps for Trp-cage, the averaged ACF profile across all time-differenced LFT features converges to zero within the maximum tested *l* = 75 (Figure S13B). The complete set of FESs and ACF curves can be found in Figures S14, S15, and S16.

**Figure 4:**
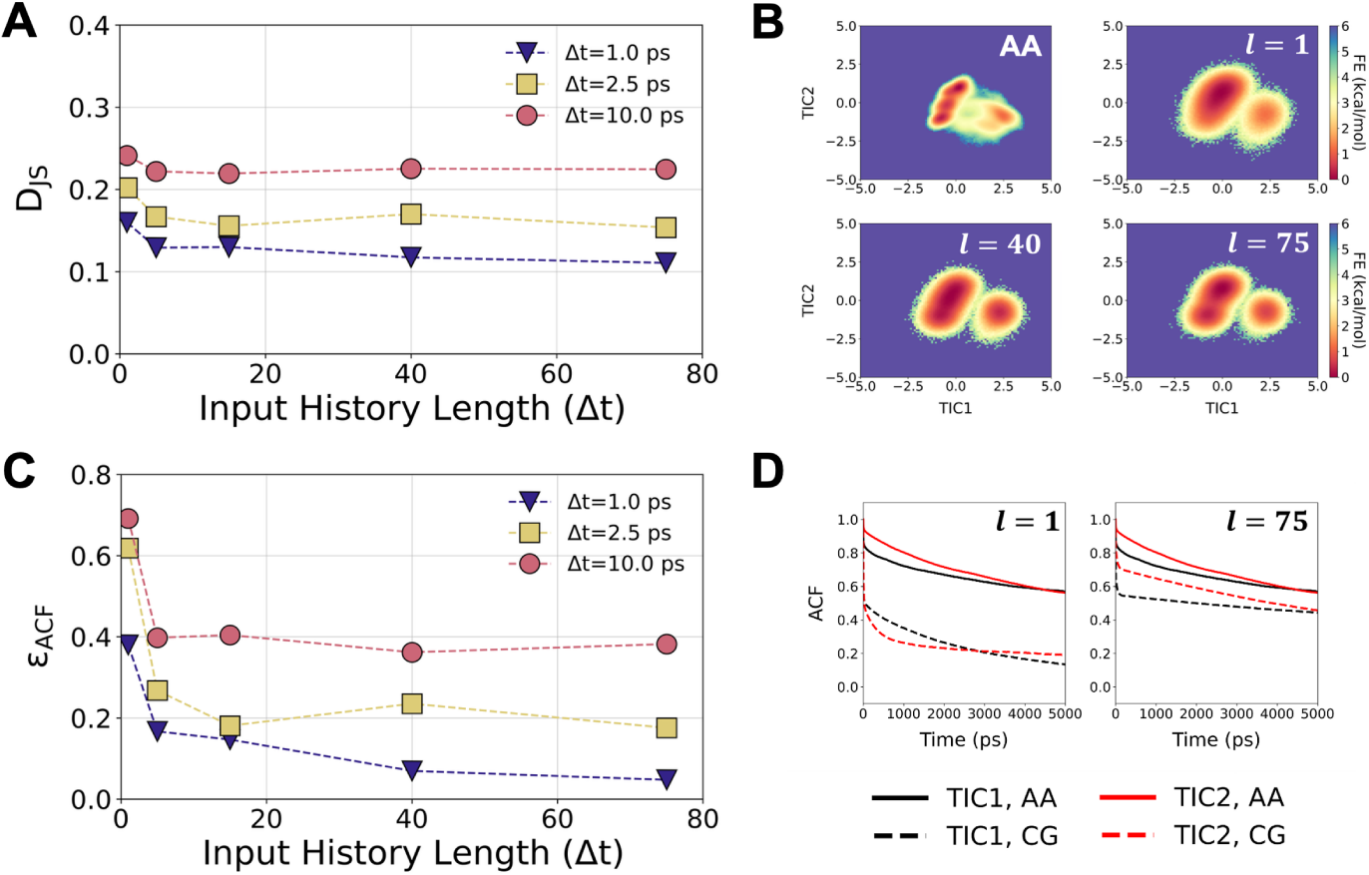
PFCG results across input history length and time step for Trp-cage. (A) Summary plot of *D_JS_* as a function of input history length, testing timesteps Δ*t* = 1.0 ps (triangle marker), 2.5 ps (square marker), and 10.0 ps (circle marker). (B) 2D FES of TIC1 and TIC2 from AA MD (top left), PFCG models at Δ*t* = 1.0 ps with *l* = 1 (top right), *l* = 40 (bottom left), and *l* = 75 (bottom right). (C) Summary plot of *ϵ_ACF_* as a function of input history length across tested timesteps. (D) Comparison of ACF curves of TIC1 and TIC2 calculated from AA MD (left) and PFCG at Δ*t* = 1.0 ps with *l* = 75 (right).

For Trp-cage, we find that increasing *l* leads to a reduction in *D_JS_* between AA and PFCG 2D TICA probability distributions, as shown in Figure 4A. We also find that increasing Δ*t* results in a systematic increase in *D_JS_*, which we attribute to the loss of fine detail in the FES. As seen in Figure 4B, the AA MD FES of Trp-cage has four metastable states, where three are clustered between −1.53 < TIC1 < 0.89 and −1.90 < TIC2 < 1.92 and the fourth is located at (TIC1, TIC2) = (2.45, −0.85). Our AA MD simulations do not undergo significant configurational changes, so the different minima represent minor fluctuations made by Trp-cage during the simulation. While both the Markovian and non-Markovian models for Δ*t* = 1.0 ps qualitatively capture the AA MD FES by distinguishing the fourth minima from the first three, both also lose detail about the first three states (see Figure 4B). For *l* = 1, we see that the three clustered minima are combined into a single basin, leading to two total minima in the FES. For the non-Markovian models with *l* = 40 and *l* = 75, we see that multiple minima are starting to be differentiated, although the energy barrier between the three states is still underestimated. The improvement in state differentiation results in the observed decrease in *D_JS_* between *l* = 1 and *l* = 75 at Δ*t* = 1.0 ps, with values of 0.16 and 0.11, respectively.

When analyzing dynamics, we find that Trp-cage CG dynamics improve with increasing *l* (as seen in Figure 4C), similarly to chignolin, with a striking decrease of *ϵ_ACF_* observed with any addition of non-Markovian information. We note that unlike chignolin, Trp-cage performance is impacted by time step and requires smaller timesteps. For instance, a time step of Δ*t* = 10.0 ps is too large with the current model for Trp-cage. We speculate that performance plateaus after *l* = 15 as the ACF of time-differenced features at Δ*t* = 10.0 ps decorrelates within a lag of 27 time-lagged points, such that increasing input history does not benefit dynamical performance (Figure S13B). While there is an improvement in *ϵ_ACF_* in reference to the Markovian model, *ϵ_ACF_* remains high (as does *D_JS_*) across all values of *l*. Even at a lower time step of Δ*t* = 2.5 ps, both *ϵ_ACF_* and *D_JS_* remain high across *l*. The ACF profiles for TIC1 and TIC2 at Δ*t* = 1.0 ps for *l* = 1 and *l* = 75 are compared in Figure 4D. For *l* = 1, faster decorrelation of TIC1 and TIC2 in CG MD is observed in comparison to that of AA MD, while TIC1 is surprisingly (and incorrectly) observed to decorrelate faster than TIC2 in CG MD. In comparison, the ACF curves for *l* = 75 show slower dynamics than the Markovian model, and the profiles of the curves are qualitatively similar to that of AA MD. While the CG dynamics still experience some acceleration at *l* = 75, notably in the initial drop of the ACF curve, the dynamics are improved in comparison to the Markovian model with *ϵ_ACF_* decreasing from 0.38 for *l* = 1 to 0.05 for *l* = 75.

### Ablation Studies

Next, we consider the importance of both model architecture elements and data sampling to PFCG performance. To assess the former, we analyzed model performances for both chignolin and Trp-cage at a time step of Δ*t* = 1.0 ps, looking at both *l* = 1 and *l* = 75. Model hyperparameters were kept constant between original models and ablation models, other than the removal of specific components from the model architecture. A summary of *D_JS_* and *ϵ_ACF_* is shown in Table 1.

**Table 1:** Ablation Study for Chignolin and Trp-cage Assessed Using 2D *D_JS_* and *ϵ_ACF_* ^a^

	Chignolin		Trp-cage	
Condition	D_JS_	*ϵ_ACF_*	D_JS_	*ϵ_ACF_*
*l* = 1, Baseline	0.066	0.213	0.164	0.412
*l* = 75, Baseline	0.107	0.025	0.111	0.048
*l* = 1,*σ_g_* = 0.0	0.088	0.211	0.243	0.662
*l* = 75,*σ_g_* = 0.0	0.094	0.129	0.238	0.517
*l* = 1, Nonprobabilistic	0.676	0.996	0.648	0.216
*l* = 75, Nonprobabilistic	0.675	0.975	0.632	0.215
*l* = 75, non-Markovian block only	0.130	0.031	0.248	0.914

a All experiments used a timestep of Δ*t* = 1.0 ps and the listed *l*, with all other model hyperparameters being the same as the respective baseline model (i.e., the reference PFCG model) aside from the ablation condition.

To investigate the impact of regularization on model performance, we removed the added Gaussian noise (originally 0.01 for chignolin and 0.02 for Trp-cage). For both chignolin and Trp-cage, the removal of Gaussian noise resulted in worse *D_JS_* and *ϵ_ACF_* values for both Markovian and non-Markovian models in comparison to the baseline PFCG models. By omitting regularization, the model is susceptible to overfitting to the noisy input trajectory data. Configurational recapitulation to atomistic data is worse for both chignolin (Figure S17A-B) and Trp-cage (Figure S18A-B), resulting in overestimation of some states and underestimation of other states. More importantly, the dynamics of large-scale configurational changes appears to be suppressed, as evident by the plateauing behavior observed in the ACF profiles.

To test the importance of the probabilistic approach, we changed the PFCG architecture to a nonprobabilistic model where the final output is explicitly *R_t_*_+1_. We used the MSE as the loss function and all other model hyperparameters were kept consistent with the original (or baseline) PFCG model (hyperparameters shown in Table S5). We find for both chignolin (Figure S17C–D) and Trp-cage (Figure S18C–D) that both the Markovian and non-Markovian nonprobabilistic models result in CG MD simulations that quickly become “pinned” (within a few integration steps) to specific configurations and do not explore phase space. The resulting *D_JS_* reflects this poor performance and is close to the upper bound of ln(2) for both systems. The plateau of the ACF profiles also reflects stagnated dynamics, where the simulations are only able to sample a specific configuration after becoming “pinned”. We interpret the failure mode of the nonprobabilistic models as a form of “mode collapse” that is mitigated by the built-in uncertainty that is learned using the original probabilistic approach.

To test the need for the pretrained Markovian block, we trained PFCG models using only the non-Markovian block to learn both spatial and temporal information simultaneously. We find that using only a non-Markovian block results in decreased *D_JS_* and *ϵ_ACF_* values in comparison the baseline PFCG model, both at *l* = 75. The FESs for chignolin (Figure S17E) and Trp-cage (Figure S18E) show multiple minima blurring into a single minimum. The blurring of minima is particularly detrimental to the chignolin model, where the folded state is significantly under sampled. The inability to represent configurational statistics propagates to simulation dynamics and shows that splitting the architecture into dedicated “effective force” (Markovian block) and “effective memory kernel” (non-Markovian block) contributions is necessary for robust model performance.

Finally, we explored the ability of the PFCG model to either interpolate or extrapolate to unseen phase space under limited data conditions. We focus these studies on chignolin, as Trp-cage does not undergo major conformational changes. Here, we discuss models with a time step of Δ*t* = 1.0 ps with *l* = 75, shown in Figure 5, and include the corresponding *l* = 1 models in Figure S19.

**Figure 5:**
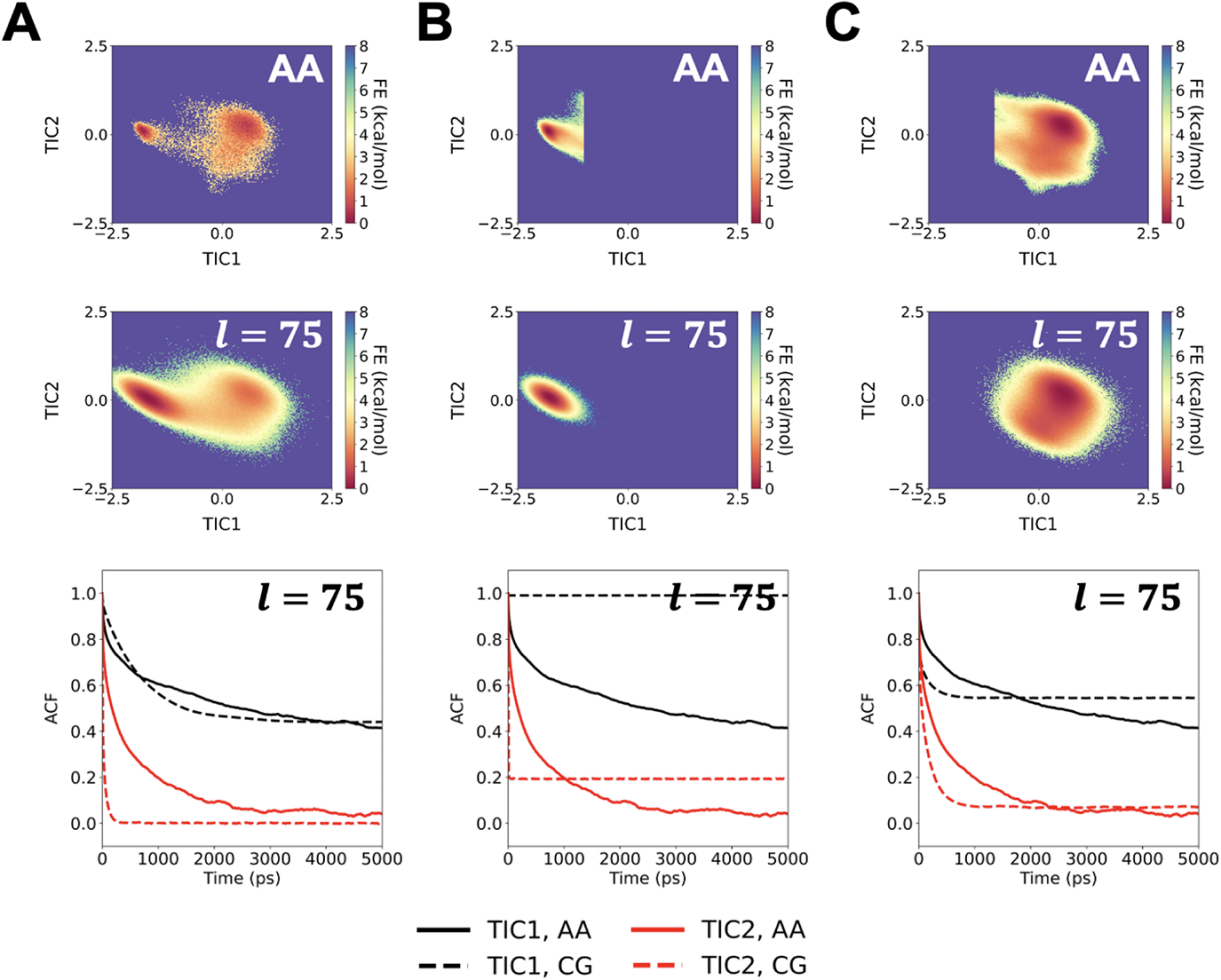
Chignolin PFCG results under limited data conditions at Δ*t =* 1.0 ps with *l* = 75. (A-C) Comparison of training data FESs (top), PFCG generated FESs (middle), and comparison of TIC1 and TIC2 ACF curves calculated from AA MD and PFCG (bottom) under conditions of (A) sparse training data, (B) training data where only the folded state is sampled, and (C) training data where only the unfolded state is sampled.

To assess the ability of the PFCG model to interpolate within phase space, we downsampled the chignolin training data set by a factor of 500, leaving a sparse FES as seen in the top panel of Figure 5A. We find that when chignolin training data is sparse but includes examples of both folded and unfolded states, the PFCG model can interpolate within the poorly sampled phase space and recapitulate the complete atomistic FES. Similarly to our prior chignolin PFCG models using the complete training data set, adding non-Markovian information with *l* = 75 improves model performance in terms of both configurational statistics and dynamics in comparison to the *l* = 1 model (Figure S19A). However, we do observe a larger 2D JSD (0.214) compared to the model trained with the full data set (0.107), which is largely attributed to the underestimated density for the unfolded state. Nonetheless, in our analysis of model dynamics, we find that the ACF profile for TIC1 is consistent with that of AA MD statistics, while the decorrelation time for TIC2 is still too rapid; the mismatched TIC2 dynamics ultimately result in a larger *ϵ_ACF_* of 0.158 compared to the baseline model (0.025).

To test whether the PFCG model can extrapolate to unseen phase space, we evaluated performance under two conditions, one where only the folded phase space is sampled in the training data (see Figure 5B), and another where only the unfolded phase space is sampled in the training data (see Figure 5C). We used a TIC1 value of −1.0 as the boundary between the folded and unfolded states. We find that under both conditions, the PFCG model fails to learn the underlying physics that would be required to extrapolate to the omitted state. When only the folded state is present in the training data, the non-Markovian model (*l* = 75) recapitulates the training data FES as shown in Figure 5B, but fails to sample the unfolded state, leading to a large 2D JSD of 0.482 in reference to the full training data set. Similarly, when the training data only samples the unfolded state, the PFCG model is unable to extrapolate to the folded state as we show in Figure 5C. In this case, the 2D JSD is 0.131 in reference to the full data set, and we note that this 2D JSD is lower than that of the model trained using the sparse data set due to the accumulated density mismatch in the sparse model in both folded and unfolded regions of the FES (i.e., the model trained using only unfolded state data recapitulates that part of phase space and only misses the folded state density). We also see that by omitting major configurational states from training, the capacity to learn complete dynamics is also removed, as observed in the ACF profiles for both *l* = 75 (Figure 5B and C) and *l* = 1 (Figure S19B and C).

In summary, the current PFCG model is able to interpolate within a sparsely sampled phase space and learn the underlying atomistic dynamics but is unable to extrapolate to states not observed in the training data. We note that extrapolating unseen states is a limitation in existing CG machine-learning approaches^35,66^ and the possibility of emergent or extrapolatory prediction from probabilistic molecular dynamics approaches remains an open question.^67^

### Summary of Mini-Protein Study

In the cases of both chignolin and Trp-cage, we find that implicitly learning *H_t_* via a history of previous configurations up to a length *l* = 75 improves the fidelity of PFCG integration compared to Markovian representations. In addition, PFCG integration is amenable to timesteps on the order of ps, which is much larger than conventional CG timesteps on the order of 10–25 fs.^5,68^ We find that the chignolin PFCG model is able to achieve larger integration timesteps (up to 10.0 ps), while still recapitulating AA MD dynamics, compared to that of Trp-cage (up to 1.0 ps). For chignolin, we find that the PFCG model can learn to interpolate within a poorly sampled phase space and partially recover dynamics (limited to the slowest mode for chignolin), although is unable to extrapolate to unseen states. To further investigate the impact of the Δ*t* and *l*, we next investigate polyalanine peptides of varying length, where the length positively correlates with the time scale for helix–coil transitions and the degree of helicity.^56^

### Impact of Polyalanine Chain Lengths on PFCG Model Fidelity

We investigated two polyalanine systems – A_5_ and A_12_ – as a means to test PFCG model fidelity on peptides where increasing the chain length leads to increased average helicity and increased helix–coil transition time scales.^55,56,69^ Given the seeming importance of decorrelation time scales on PFCG model performance, we posit that larger polyalanine chains may perform better either with increasing non-Markovian sequence lengths or larger timesteps. Indeed, the average ACF across all time-differenced features (see Figure S20) show that the minimum Δ*t* required to observe decorrelation within 100 time-lagged steps is 1.0 and 10.0 ps for A_5_ and A_12_, respectively. To test this idea, AA MD was performed for each polyalanine peptide then coarse-grained to one CG site per residue and LFT featurized. Markovian models were optimized first to find both model architecture hyperparameters and Δ*t*. Unlike in the case of chignolin and Trp-cage, we found that exploration of the partially helical states of polyalanine to be dependent on Δ*t*. After comparing different Δ*t* values, we found that Δ*t* = 1.0 ps and Δ*t* = 10.0 ps for A_5_ and A_12_, respectively, ensured proper exploration; an example of poor exploration due to using too small of a Δ*t* is shown in Figure S21, although we suspect that increasing model capacity (i.e., increasing *N_MHA_*___ *_MLP_*) may mitigate this issue as we performed hyperparameter tuning in a small search space for computational efficiency. Non-Markovian models were trained using the same hyperparameters from the Markovian models with *l* = 1, 5, 10, 40, 60, and 100 as *l* = 100 is our practical upper limit for A_15_ due to memory limitations. All optimized model hyperparameters are found in Table S5. The polyalanine models were assessed similarly to the miniproteins, although *ϵ*_*ACF*_ was computed using only TIC1 as this component best aligned with the helix–coil transition dynamics. To obtain a more holistic picture of the system dynamics, two-state MSMs were generated from all fitted TICA components with MFPTs assessed. When considering the totality of *D_JS_*, *ϵ_ACF_*, and *MARE_MFPT_*, we determined that the “optimal” *l* is *l* = 40 for A_5_ with Δ*t* = 1.0 ps (40 ps total history) and *l* = 10 for A_12_ with Δ*t* = 10.0 ps (100 ps total history). Below, we will specifically focus on these cases as the best performing non-Markovian models.

The polyalanine PFCG models generally reproduce the dominant features of the 2D AA FESs, such as basins corresponding to helical, partially helical, and random coil states (see Figure 6A-B). For example, both the AA and PFCG FESs for A_5_ (Figure 6A) exhibit minima corresponding to the helical and random coil states, with (TIC1,TIC2) = (2.3,0.2) and (−0.4,0.05), respectively. In the AA model, the random coil state is favored by approximately 1.6 kcal/mol relative to the helical state. The corresponding free energy differences predicted by the Markovian (*l* = 1) and non-Markovian (*l* = 40) PFCG models are around 1.0 and 1.2 kcal/mol, respectively, indicating reasonable quantitative agreement in relative state stability.

**Figure 6:**
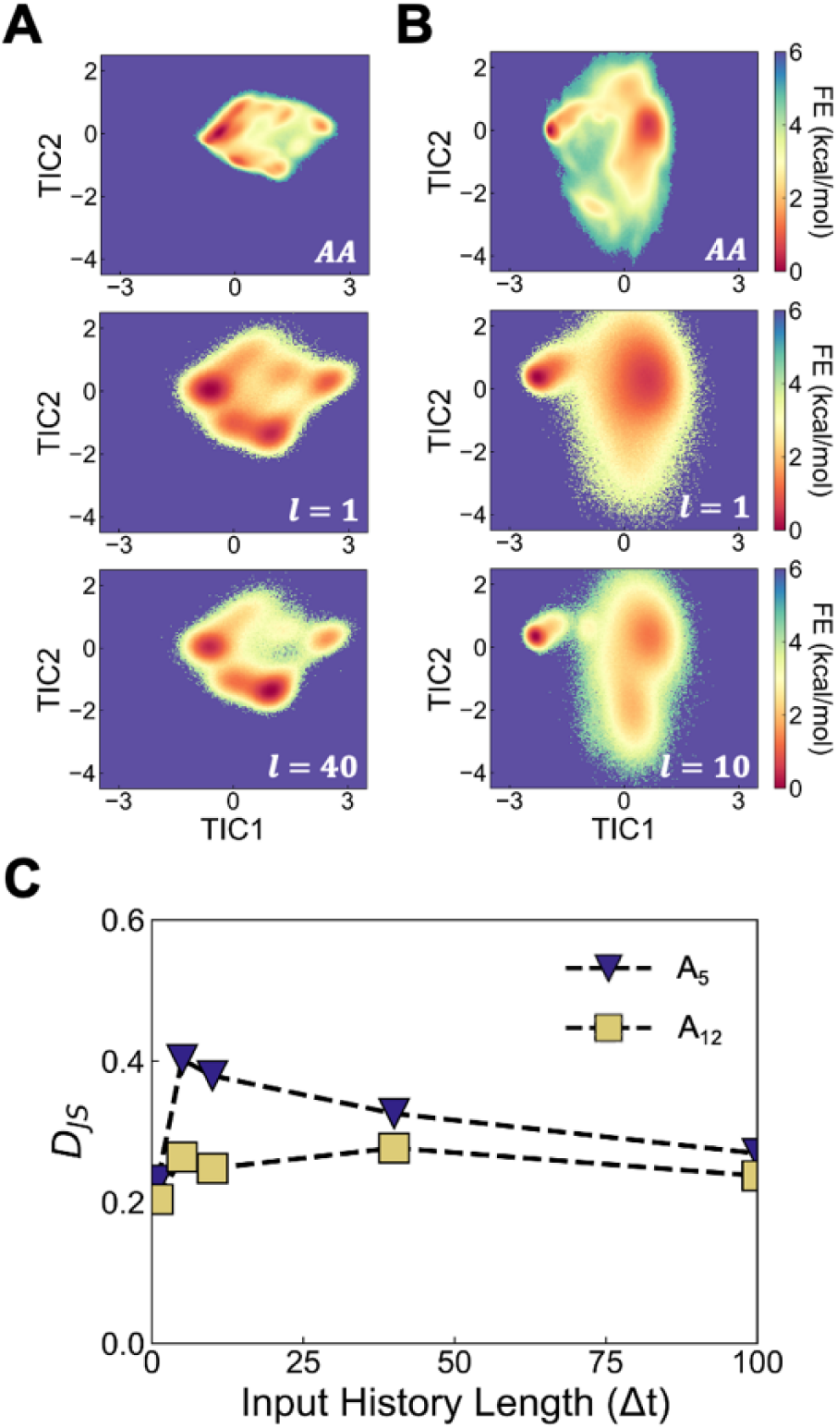
Thermodynamic consistency of polyalanine PFCG models. (A-B) Comparison of free energy (FE) surfaces from AAMD, Markovian PFCG, and non-Markovian PFCG models for (A) A_5_ and (B) A_12_. (C) Comparison of *D_JS_* as the input history length (*l*) is varied for both A_5_ (blue triangles) and A_12_ (yellow squares).

For A_5_, inclusion of non-Markovian history primarily affects the population of intermediate configurations rather than the overall topology of the FES. In the *l* = 40 PFCG model, the partially helical states centered around (1.1,-1.0) and (1.3, 0.5) are overrepresented and underrepresented, respectively, relative to the *l* = 1 PFCG model, but the locations of the helical and random coil minima remain unchanged. As a result, as seen in Figure 6C, the inclusion of history slightly increases *D_JS_* relative to the Markovian baseline (*D_JS_* = 0.230) with average values of 0.322±0.063 across non-Markovian models.

In contrast, A_12_ (Figure 6B) exhibits a broader and more structured helix–coil transition pathway in the AA FES, consistent with its longer chain length and slower transition dynamics. The helix–coil transition spans a continuous pathway from a helical state centered near (−1.9,0.0) to a broad random coil basin near (0.7,0.0). The PFCG models predict an offset helical state centered near (−2.2,0.4) instead, which reflects longer bond distances in the C-terminus atoms compared to atomistic data. In addition, while the Markovian *l* = 1 model captures the dominant helical and random coil basins, it fails to resolve an intermediate partially helical state near (−0.7,0.4) while the random coil basin is overly diffuse. Incorporation of non-Markovian history (e.g., *l* = 10) recovers this intermediate basin and yields a helix–coil transition pathway that more closely resembles the topology of the AA FES. Despite these qualitative improvements, the non-Markovian models do not consistently reduce *D_JS_* relative to the Markovian baseline, with average values increasing from 0.204 (*l* = 1) to 0.273±0.037 across non-Markovian models. We attribute this observation to the fact that *D_JS_* heavily penalizes the absence of density compared to differences in the fine structure of the density, and the Markovian model ensures all relevant parts of phase space are sampled even if intermediate states are overpopulated. Finally, we note that the average increase in *D_JS_* upon adding history is smaller for A_12_ (around 0.069) than for A_5_ (0.092), suggesting that history-dependent modeling is less detrimental for configurational sampling for systems with slower, more distributed transition pathways. This trend is consistent with the observation that non-Markovian models for A_12_ introduce additional intermediate states rather than redistributing probability density among existing basins.

Dynamical fidelity was assessed using ACFs of TIC1, which most directly reflects helix–coil transitions (Figure 7A-B). For both A_5_ and A_12_, the non-Markovian models reduce deviations from AA decorrelation times relative to the Markovian baseline. This trend extends to improvements over traditional CG approaches such as force matching on pairwise interactions (Figure S22). For A_5_, the impact of history is nonmonotonic where 5 ≤ *l* ≤ 40 results in ACF profiles comparable to that of AA, while larger *l* lead to faster-than-AA decorrelations. Similarly, A_12_ exhibits consistently improved dynamical agreement across all tested non-Markovian models, with *ϵ_ACF_* < 0.005 when 5 ≤ *l* ≤ 40, compared to *ϵ_ACF_* = 0.027 for the Markovian model (Figure 7B). These results suggest that the inclusion of too much history may lead to loss in model performance, which we speculate is more from accumulation of “integration” error of *H_t_* across the sequence *R*_*t*–*l*+1_ to *R_t_* during inference. In the future, it may be necessary to include an exponentially decaying weighting factor or alternate regularization technique to mitigate possible integration errors from long trajectory histories.

**Figure 7:**
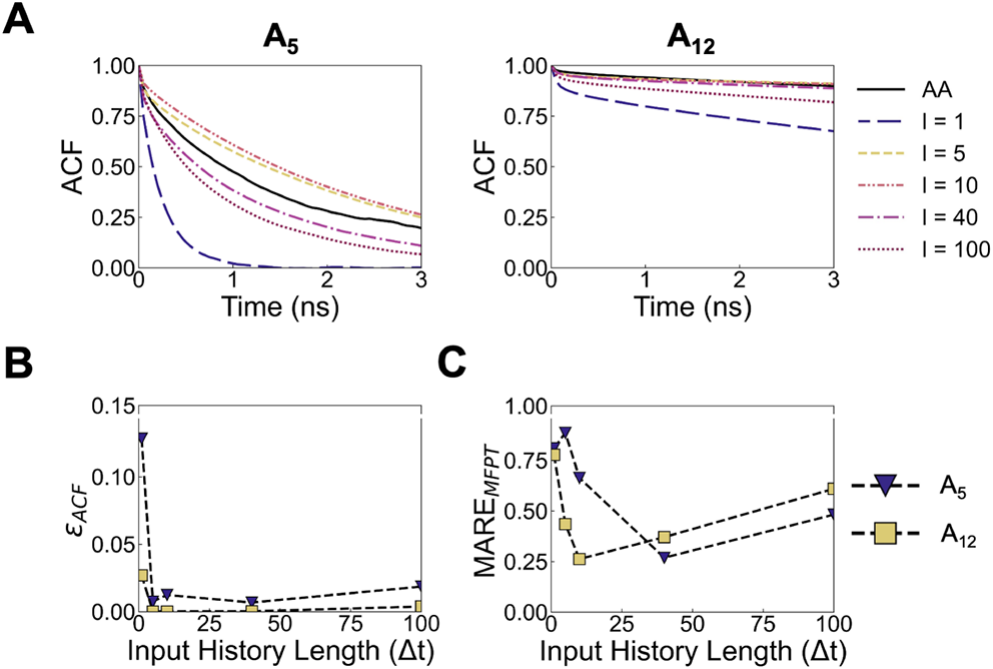
Dynamical consistency of polyalanine PFCG models. (A) Comparison of autocorrelation functions (ACFs) from AA (solid line) and PFCG (dashed lines) MD simulations using the listed input history lengths (*l*). (B) Summary plot of TIC1 *ϵ_ACF_* as a function of input history length for A_5_ and A_12_. (C) Mean absolute relative error of the mean first passage time (*MARE_MFPT_*) between the AA and PFCG models as a function of input history length for A_5_ and A_12_.

Additional insight into transition kinetics is provided by two-state MSMs constructed from TICA coordinates trained from AA trajectories and applied to PFCG trajectories (Table 2 and Figure S23). For both A_5_ and A_12_, the best performing non-Markovian models reduce MPFT errors relative to Markovian models for helix–coil transitions. For A_5_, the non-Markovian (*l* = 40) model reduces MFPT percent errors from 75% to 35% (for helix to coil) and from 87% to 19% (for coil to helix), with greater improvement corresponding to the slower transition of the two. For A_12_, the MFPT error decreases from approximately 78% in the Markovian model to around 26% in the non-Markovian model (*l* = 10). These improvements indicate that the non-Markovian PFCG models better capture the relative time scales of helix–coil transitions even if the stationary distributions are largely unaffected.

**Table 2:** Comparison of Mean First Passage Times (MFPT) and the Percent Error (%err) from Markov State Models Using Two PCCA+ Clusters, Trained from Atomistic TICA Statistics for A_5_ and A_12_ and Applied to PFCG Simulations^a^

System	MFPT_0**→**1_ (%err)	MFPT_1**→**0_ (%err)
**A**_**5**_		
AA	0.89	19.55
*l* = 1	0.22 (75%)	2.52 (87%)
*l* = 40	0.58 (35%)	15.92 (19%)
**A**_**12**_		
AA	12.87	47.61
*l* = 1	2.88 (78%)	10.48 (78%)
*l* = 10	16.42 (27%)	35.74 (25%)

a States 0 and 1 refer to the helical and random coil states, respectively.

The variation in MSM-based time scale accuracy with *l*, quantified by MARE_MFPT_ (Figure 7C), broadly reflects the TIC1-based *ϵ_ACF_* (Figure 7B), indicating that both metrics capture related aspects of dynamical fidelity. However, the two measures probe different projections of dynamics, where *ϵ_ACF_* captures decorrelation along a single slow mode (TIC1) while the MFPTs from the MSMs capture slow modes that can occur in higher dimensional TIC space. As a result, MSM-based errors are more sensitive to the treatment of intermediate states that span macrostate cluster boundaries, particularly in A_12_ where helix–coil transitions proceed through multiple partially helical intermediates. We therefore observe larger discrepancies between MSM and ACF metrics for A_12_ than for A_5_, even when single-mode decorrelation is well reproduced. Despite these differences, the non-Markovian PFCG models consistently improve dynamical agreement relative to Markovian models across both metrics.

The choice of time step (Δ*t*) and input history length (*l*) reflects a coupled trade-off between physical time scale coverage and numerical stability. The slowest AA time scales extracted from MSMs are approximately 19.55 ns for A_5_ and 47.61 ns for A_12_, consistent with the increased history required to model A_12_ dynamics (100 ps of history) compared to A_5_ dynamics (40 ps of history). However, optimal performance does not correspond to simply increasing *l* at fixed Δ*t*. Increasing *l* introduces challenges associated with long-sequence integration in GRU-based and other recurrent neural network architectures (such as the vanishing gradient problem, although alleviated by the GRU architecture), particularly as *h_dim_* increases, while increasing Δ*t* to reduce *l* is limited by integration error that degrades faster motions. Consequently, we find that Δ*t* and *l* cannot be tuned independently. Larger systems with slower transitions (e.g., A_12_ compared to A_5_) require balancing longer effective history against stability constraints imposed by both the integrator and the recurrent model. Resolving this trade-off will be critical for extending PFCG models to larger and more complex biomolecular systems, which may require changes to model architectures or training methodologies.

### Computational Efficiency of PFCG

We tested the computational efficiency of PFCG MD simulations in comparison to AA MD of trained PFCG models excluding training time. Training for PFCG took between 3 to 12 h on a single A100 GPU depending on the stage of training (Markovian vs non-Markovian), the size of the PFCG model architecture, training data set size, and miniprotein system. For PFCG simulations, we computed the throughput of Markovian models and the best performing non-Markovian models on a single A100 GPU. As seen in Table 3, the throughput (in ns/day) of both the miniprotein and polyalanine systems is much greater in comparison to AA MD by at least an order of magnitude on a per-replica basis. In reality, the throughput is increased by 3 orders of magnitude as we are comfortably able to simulate 128 PFCG replicas at once. In addition, we find that the non-Markovian models simulate at nearly half the speed of corresponding Markovian models for all systems, which is unsurprising as the Markovian models are nearly half the size of their corresponding non-Markovian models.

**Table 3:** Computational Throughput of AA and PFCG MD Simulations When Using an A100 GPU (in ns/day/replica)

Method	Δ*t* (ps)	*l*	Replica	Rate (ns/day/replica)
**Chignolin**				
AA MD	0.002		1	485
PFCG MD	1.0	1	128	4,960
PFCG MD	1.0	75	128	2,670
**Trp-cage**				
AA MD	0.002		1	500
PFCG MD	1.0	1	128	5,030
PFCG MD	1.0	75	128	1,960
**A**_**5**_				
AA MD	0.002		4	120
PFCG MD	1.0	1	128	2,720
PFCG MD	1.0	40	128	1,590
**A**_**12**_				
AA MD	0.002		4	90
PFCG MD	10.0	1	128	24,400
PFCG MD	10.0	10	128	12,600

The polyalanine systems show, as expected, that throughput is linearly correlated with Δ*t* as A_12_, which has around a factor of 10 larger throughput compared to A_5_, was also run with a factor of 10 larger Δ*t*. Like with any MD simulation, the highest throughput is achieved when run with the highest reliable Δ*t*, which is limited by integration error, with the PFCG model capable of Δ*t* values far beyond that of traditional AA (1–2 fs) and CG (10–25 fs) models. Second, throughput is linearly impacted by larger *l*; for A_12_, each added frame of history to the input sequence translated to an average loss of ∼ 32 ns/day/replica over a range of 5 < *l* < 100 frames (Figure S24A). Given that this value is 0.2% of the non-Markovian throughput for A_12_, the input history length has a negligible impact on throughput. Lastly, most model architecture hyperparameters have minor impacts on simulation speed. The major exception is *N_MHA_*___ *_MLP_* as the self-attention layers are sequential (not parallelizable). We find that each added layer (up to 3 layers) results in a decrease to the throughput by 4,470 ns/day/replica for A_12_ (Figure S24B). While fewer layers are ideal, the best performing PFCG models tended to require multiple layers.

## Conclusion

In this work, we introduce Probabilistic Forecasting for Coarse-Graining (PFCG), a history-dependent, data-driven framework for learning coarse-grained equations of motion directly from atomistic molecular dynamics data. PFCG models the time evolution of coarse-grained coordinates (and learned hidden auxiliary variables) as a stochastic dynamical process, producing probabilistic, autoregressive forecasts that capture both stationary configurational statistics and dynamical correlations. By incorporating explicit temporal history via hidden auxiliary variables, PFCG provides a systematic approach for modeling non-Markovian effects that arise naturally from low-resolution, implicit-solvent coarse-graining, without requiring predefined memory kernels as one might expect from the Generalized Langevin Equation. We demonstrated our approach on benchmark miniproteins and polyalanine peptides, showing that non-Markovian PFCG models improve dynamical fidelity relative to Markovian baselines while preserving agreement with atomistic free energy landscapes.

A distinguishing feature of PFCG is the form of inductive bias imposed on the coarse-grained model. Many existing machine-learned coarse-graining approaches learn effective interaction potentials via neural networks that demonstrably reproduce atomistic structural observables.^25,27^ In these approaches, dynamical behavior may emerge implicitly through careful choice of thermostat and integrator, although systematic studies on the dynamical consistency of these methods is still lacking. In contrast, PFCG learns the dynamics directly by modeling the time-evolution of coarse-grained coordinates as a stochastic process composed of both Markovian and non-Markovian contributions. This shifts the learning objective from reproducing static distributions to approximating the underlying equations of motion governing coarse-grained dynamics, embedding temporal correlation and stochasticity directly into the model architecture. Our approach is therefore complementary to prior machine-learned methods that propagate time-evolution on a low-dimensional manifold, with atomic configurations generated from learned decoders.^34,35^ These prior methods prioritize learning a compressed latent representation (typically described by one to three latent dimensions for their chosen peptides and miniproteins) that either function in the Markovian limit with sufficiently long lag times^34^ or also use recurrent neural networks to capture non-Markovian behavior,^35^ although dynamical consistency in the latter case has only been demonstrated using a relatively simple alanine dipeptide system. PFCG instead operates under a fixed and physically interpretable coarse-grained mapping (with between 15 to 60 dimensions for our tested peptides and miniproteins) and prioritizes learning the direct coarse-grained dynamics.

Our results further highlight that improvements in dynamical fidelity do not necessarily coincide with improved agreement in stationary distributions, underscoring the importance of evaluating coarse-grained models using both configurational and kinetic metrics. Autocorrelation-based measures and MSM-derived time scales probe complementary aspects of dynamics, particularly in systems with heterogeneous transition pathways and multiple intermediate states. The observed trade-offs between time step size (Δ*t*) and input history length (*l*) reflect fundamental constraints associated with long-time scale integration, recurrent sequence modeling, and numerical stability, rather than limitations of any specific implementation. Based on analysis of ACFs for time-differenced input features (Figures S8, S13, and S20), we suggest a heuristic where *l* should be long enough to capture complete decorrelation of the ACF at a given Δ*t*. Nonetheless, we highlight that the PFCG method achieves timesteps on the order of ps, which is three (and two) orders of magnitude larger than those possible with conventional atomistic (and coarse-grained) molecular dynamics simulations.

Looking forward, several future directions emerge naturally from this work. Extending PFCG to larger and more complex biomolecular systems will require systematic strategies to navigate the aforementioned trade-offs between time step selection, memory depth, and model capacity. Architectural developments, such as graph neural networks^25,70^ that enforce invariance or equivariance, may be necessary to make PFCG more accessible to these larger systems, especially when intermolecular interactions are involved. Further, larger systems with slower time scale motions than tested here may require untenable amounts of data, although our experiments with models trained on sparse data suggest that it may be possible to mitigate data limitations to a certain extent. Another aspect is the disconnect between model evaluation during training and performance during inference as the loss only measures one-step integration performance and does not account for long-time horizon prediction. To this end, methods from the machine learning community to model seasonal forecasting^71^ may be helpful to enforce learning of slow structural transitions. Finally, one important limitation of our current approach (and other data-driven approaches, generally) is that the physics captured by the model depends on the states observed within the training data set. The current PFCG model is unable to extrapolate to configurations never seen during training. However, we hypothesize that changes to model architecture and training that focus on local interactions (e.g., graph neural networks or local basis functions trained on large sets of peptide fragments), combined with physics-informed regularization (e.g., force matching on the Markovian block) and enhanced sampling^72–74^ (e.g., umbrella sampling or metadynamics combined with reweighting) to discover all relevant examples of local interactions, is a viable path forward for PFCG models that can eventually discover emergent behavior.

One interesting future direction that leverages the current PFCG model is to formally investigate the “time acceleration” of CG models. It has long been recognized that CG time does not strictly correlate with real time, although a common heuristic is to linearly scale CG time based on the relative differences in diffusivity compared to atomistic simulations or based on the time scale of observed phenomenology compared to experiments.^75^ More recent approaches for uniform corrections to dynamics include deriving an effective CG time acceleration and friction parameter through adversarial learning^76^ or quantifying scaling relationships from excess entropy analysis.^77,78^ However, it is unlikely that such a uniform scaling truly exists in complex, heterogeneous systems as the barriers in the underlying atomistic free energy surface are likely smoothed upon coarse-graining in a nontrivial and nonuniform manner across phase space. Our construction of the PFCG model with both Markovian and non-Markovian contributions potentially offers a principled way to investigate nonuniform “time acceleration” across phase space. More broadly, PFCG provides a foundation to investigate kinetically controlled molecular processes where dynamical consistency is important, such as by incorporating external driving forces or nonequilibrium control protocols. By shifting the focus of coarse-graining from effective interactions to learned stochastic dynamics, PFCG opens a new avenue for constructing dynamically faithful coarse-grained models of complex molecular systems.

## Supplementary Material



## Data Availability

The data underlying this study, including simulation files, analysis scripts, and processed data, are openly available on GitLab: https://gitlab.com/pak-group/pfcg_m01.

## Notes

Additional details on simulation conditions and data on free energy surfaces, autocorrelation functions, Markov state model training, and computational throughput analysis (PDF)

## References

[1] Hollingsworth S. A., Dror R. O. (2018). Molecular Dynamics Simulation for All. Neuron.

[2] Perilla J. R., Goh B. C., Cassidy C. K., Liu B., Bernardi R. C., Rudack T., Yu H., Wu Z., Schulten K. (2015). Molecular
dynamics simulations of large macromolecular complexes. Curr. Opin. Struc. Biol..

[3] Park H. Y., Kim S. A., Korlach J., Rhoades E., Kwok L. W., Zipfel W. R., Waxham M. N., Webb W. W., Pollack L. (2008). Conformational
changes of calmodulin upon Ca_2+_ binding studied with a
microfluidic mixer. Proc. Natl. Acad. Sci. U.
S. A..

[4] Whitford P. C., Onuchic J. N. (2025). Simulating biomolecules for physiological timescales. Curr. Opin. Struc. Biol..

[5] Souza P. C. T., Alessandri R., Barnoud J., Thallmair S., Faustino I., Grunewald F., Patmanidis I., Abdizadeh H., Bruininks B. M. H., Wassenaar T. A. (2021). Martini 3: a general purpose force field for coarse-grained molecular
dynamics. Nat. Methods.

[6] Allen E. C., Rutledge G. C. (2008). A novel algorithm
for creating coarse-grained, density
dependent implicit solvent models. J. Chem.
Phys..

[7] Lyubartsev A. P., Laaksonen A. (1995). Calculation
of Effective Interaction Potentials from
Radial-Distribution Functions - a Reverse Monte-Carlo Approach. Phys. Rev. E.

[8] Reith D., Putz M., Muller-Plathe F. (2003). Deriving effective
mesoscale potentials
from atomistic simulations. J. Comput. Chem..

[9] Hills R. D., Lu L., Voth G. A. (2010). Multiscale coarse-graining
of the protein energy landscape. PLoS Comput.
Biol..

[10] Chaimovich A., Shell M. S. (2011). Coarse-graining errors and numerical optimization using
a relative entropy framework. J. Chem. Phys..

[11] Izvekov S., Voth G. A. (2005). A Multiscale Coarse-Graining
Method for Biomolecular
Systems. J. Phys. Chem. B.

[12] Noid W. G., Chu J.-W., Ayton G. S., Krishna V., Izvekov S., Voth G. A., Das A., Andersen H. C. (2008). The multiscale coarse-graining
method. I. A rigorous bridge between atomistic and coarse-grained
models. J. Chem. Phys..

[13] Noid W. G., Liu P., Wang Y., Chu J. W., Ayton G. S., Izvekov S., Andersen H. C., Voth G. A. (2008). The multiscale coarse-graining method.
II. Numerical implementation for coarse-grained molecular models. J. Chem. Phys..

[14] Keith J. A., Vassilev-Galindo V., Cheng B. Q., Chmiela S., Gastegger M., Mueller K. R., Tkatchenko A. (2021). Combining Machine Learning and Computational
Chemistry for Predictive Insights Into Chemical Systems. Chem. Rev..

[15] Pak A. J., Gupta M., Yeager M., Voth G. A. (2022). Inositol Hexakisphosphate
(IP6) Accelerates Immature HIV-1 Gag Protein Assembly toward Kinetically
Trapped Morphologies. J. Am. Chem. Soc..

[16] Ivanov M., Lyubartsev A. P. (2024). Development of a bottom-up coarse-grained model for
interactions of lipids with TiO 2 nanoparticles. J. Comput. Chem..

[17] Qian H.-J., Carbone P., Chen X., Karimi-Varzaneh H. A., Liew C. C., Müller-Plathe F. (2008). Temperature-Transferable
Coarse-Grained Potentials for Ethylbenzene, Polystyrene, and Their
Mixtures. Macromolecules.

[18] Bayramoglu B., Faller R. (2012). Coarse-Grained Modeling
of Polystyrene in Various Environments
by Iterative Boltzmann Inversion. Macromolecules.

[19] Larini L., Lu L. Y., Voth G. A. (2010). The multiscale
coarse-graining method.
VI. Implementation of three-body coarse-grained potentials. J. Chem. Phys..

[20] Das A., Andersen H. C. (2012). The multiscale coarse-graining
method. IX. A general
method for construction of three body coarse-grained force fields. J. Chem. Phys..

[21] Jin J., Han Y. N., Voth G. A. (2019). Coarse-graining
involving virtual
sites: Centers of symmetry coarse-graining. J. Chem. Phys..

[22] Pak A. J., Dannenhoffer-Lafage T., Madsen J. J., Voth G. A. (2019). Systematic Coarse-Grained
Lipid Force Fields with Semiexplicit Solvation via Virtual Sites. J. Chem. Theory Comput..

[23] John S. T., Csányi G. (2017). Many-Body
Coarse-Grained Interactions Using Gaussian
Approximation Potentials. J. Phys. Chem. B.

[24] Majewski M., Pérez A., Thölke P., Doerr S., Charron N. E., Giorgino T., Husic B. E., Clementi C., Noé F., De Fabritiis G. (2023). Machine learning coarse-grained potentials of protein
thermodynamics. Nat. Commun..

[25] Husic B. E., Charron N. E., Lemm D., Wang J., Perez A., Majewski M., Kramer A., Chen Y. Y., Olsson S., de Fabritiis G. (2020). Coarse graining molecular
dynamics with graph
neural networks. J. Chem. Phys..

[26] Wang J., Olsson S., Wehmeyer C., Perez A., Charron N. E., de Fabritiis G., Noe F., Clementi C. (2019). Machine Learning of
Coarse-Grained Molecular Dynamics Force Fields. ACS Cent. Sci..

[27] Zhang L. F., Han J. Q., Wang H., Car R., E W. N. (2018). DeePCG:
Constructing coarse-grained models via deep neural networks. J. Chem. Phys..

[28] Unke O. T., Chmiela S., Sauceda H. E., Gastegger M., Poltavsky I., Schütt K. T., Tkatchenko A., Müller K. R. (2021). Machine Learning Force Fields. Chem. Rev..

[29] Ma L. N., Li X. T., Liu C. (2016). The derivation and
approximation
of coarse-grained dynamics from Langevin dynamics. J. Chem. Phys..

[30] Kinjo T., Hyodo S. A. (2007). Equation of motion
for coarse-grained simulation based
on microscopic description. Phys. Rev. E.

[31] Klippenstein V., Tripathy M., Jung G., Schmid F., van der
Vegt N. F. A. (2021). Introducing Memory in Coarse-Grained Molecular Simulations. J. Phys. Chem. B.

[32] Kadupitiya J. C. S., Fox G. C., Jadhao V. (2022). Solving Newton’s
equations
of motion with large timesteps using recurrent neural networks based
operators. Mach. Learn.: Sci. Technol..

[33] Tsai S.-T., Kuo E.-J., Tiwary P. (2020). Learning molecular
dynamics with
simple language model built upon long short-term memory neural network. Nat. Commun..

[34] Sidky H., Chen W., Ferguson A. L. (2020). Molecular latent
space simulators. Chem. Sci..

[35] Vlachas P. R., Zavadlav J., Praprotnik M., Koumoutsakos P. (2022). Accelerated
Simulations of Molecular Systems through Learning of Effective Dynamics. J. Chem. Theory Comput..

[36] Ceriotti M., Bussi G., Parrinello M. (2010). Colored-Noise
Thermostats a la Carte. J. Chem. Theory Comput..

[37] Li Z., Lee H. S., Darve E., Karniadakis G. E. (2017). Computing
the non-Markovian coarse-grained interactions derived from the Mori-Zwanzig
formalism in molecular systems: Application to polymer melts. J. Chem. Phys..

[38] Wang J., Ferguson A. L. (2018). Recovery of protein
folding funnels from single-molecule
time series by delay embeddings and manifold learning. J. Phys. Chem. B.

[39] Topel M., Ferguson A. L. (2020). Reconstruction of
protein structures from single-molecule
time series. J. Chem. Phys..

[40] Yu Y., Si X., Hu C., Zhang J. (2019). A review of recurrent neural networks:
LSTM cells and network architectures. Neural
Comput..

[41] Noid W. G. (2023). Perspective:
Advances, Challenges, and Insight for Predictive Coarse-Grained Models. J. Phys. Chem. B.

[42] Teutsch, P. ; Mäder, P. Flipped classroom: effective teaching for time series forecasting. arXiv arXiv:2210.08959. 2022.

[43] Vaswani, A. ; Shazeer, N. ; Parmar, N. ; Uszkoreit, J. ; Jones, L. ; Gomez, A. N. ; Kaiser, Ł. ; Polosukhin, I. Attention is all you need 31st Conference on Neural Information Processing Systems (NIPS 2017), Long Beach, CA, USA 2017

[44] Ba, J. L. ; Kiros, J. R. ; Hinton, G. E. Layer normalization. arXiv arXiv:1607.06450. 2016.

[45] Chung, J. ; Gulcehre, C. ; Cho, K. ; Bengio, Y. Empirical evaluation of gated recurrent neural networks on sequence modeling. arXiv arXiv:1412.3555. 2014.

[46] Honda S., Yamasaki K., Sawada Y., Morii H. (2004). 10 residue folded peptide
designed by segment statistics. Structure.

[47] Neidigh J. W., Fesinmeyer R. M., Andersen N. H. (2002). Designing a 20-residue protein. Nat. Struct. Biol..

[48] Tien M. Z., Sydykova D. K., Meyer A. G., Wilke C. O. (2013). PeptideBuilder:
A simple Python library to generate model peptides. Peerj.

[49] Huang J., Rauscher S., Nawrocki G., Ran T., Feig M., de Groot B. L., Grubmuller H., MacKerell A. D. (2017). CHARMM36m: An improved force field
for folded and intrinsically
disordered proteins. Nat. Methods.

[50] Lu J. B., Qiu Y. Q., Baron R., Molinero V. (2014). Coarse-Graining of
TIP4P/2005, TIP4P-Ew, SPC/E, and TIP3P to Monatomic Anisotropic Water
Models Using Relative Entropy Minimization. J. Chem. Theory Comput..

[51] Abraham M. J., Murtola T., Schulz R., Páll S., Smith J. C., Hess B., Lindahl E. (2015). GROMACS: High
performance
molecular simulations through multi-level parallelism from laptops
to supercomputers. SoftwareX.

[52] Bussi G., Zykova-Timan T., Parrinello M. (2009). Isothermal-isobaric molecular dynamics
using stochastic velocity rescaling. J. Chem.
Phys..

[53] Martoňák R., Laio A., Parrinello M. (2003). Predicting crystal structures: the
Parrinello-Rahman method revisited. Phys. Rev.
Lett..

[54] Chen X. N., Liang C., Huang D., Real E., Wang K. Y., Pham H., Dong X. Y., Luong T., Hsieh C. J., Lu Y. F. (2023). Symbolic Discovery of Optimization Algorithms. Adv. Neural Inf. Process. Syst..

[55] Raucci R., Colonna G., Castello G., Costantini S. (2013). Peptide folding
problem: A molecular dynamics study on polyalanines using different
force fields. Int. J. Pept. Res. Ther..

[56] Kuczera K., Szoszkiewicz R., He J., Jas G. S. (2021). Length Dependent
Folding Kinetics of Alanine-Based Helical Peptides from Optimal Dimensionality
Reduction. Life.

[57] Röblitz S., Weber M. (2013). Fuzzy spectral clustering by PCCA+: application to Markov state models
and data classification. Adv. Data Anal. Classif..

[58] Wu H., Noé F. (2020). Variational
approach for learning Markov processes
from time series data. J. Nonlinear Sci..

[59] Van Kampen, N. G. Stochastic processes in physics and chemistry; Elsevier, 1992.

[60] Hoffmann M., Scherer M., Hempel T., Mardt A., de Silva B., Husic B. E., Klus S., Wu H., Kutz N., Brunton S. L. (2022). Deeptime: a Python library
for machine learning
dynamical models from time series data. Mach.
Learn. Sci. Technol..

[61] Maruyama Y., Mitsutake A. (2018). Analysis of Structural Stability
of Chignolin. J. Phys. Chem. B.

[62] Sobieraj M., Setny P. (2022). Granger Causality Analysis of Chignolin Folding. J. Chem. Theory Comput..

[63] Byrne A., Williams D. V., Barua B., Hagen S. J., Kier B. L., Andersen N. H. (2014). Folding Dynamics
and Pathways of the Trp-Cage Miniproteins. Biochemistry.

[64] Kim S. B., Palmer J. C., Debenedetti P. G. (2016). Computational
investigation of cold
denaturation in the Trp-cage miniprotein. Proc.
Natl. Acad. Sci. U. S. A..

[65] Chen Y. Y., Krämer A., Charron N. E., Husic B. E., Clementi C., Noé F. (2021). Machine learning
implicit solvation for molecular dynamics. J.
Chem. Phys..

[66] Wu, H. ; Mardt, A. ; Pasquali, L. ; Noe, F. Deep Generative Markov State Models 32nd Conference on Neural Information Processing Systems (NeurIPS 2018), Montréal, Canada 2018

[67] Olsson S. (2026). Generative
molecular dynamics. Curr. Opin. Struc. Biol..

[68] Klein F., Sonora M., Santos L. H., Frigini E. N., Ballesteros-Casallas A., Machado M. R., Pantano S. (2023). The SIRAH force field: A suite for
simulations of complex biological systems at the coarse-grained and
multiscale levels. J. Struct. Biol..

[69] Wu J., Zhen X., Shen H. J., Li G. H., Ren P. Y. (2011). Gay-Berne
and electrostatic multipole based coarse-grain potential in implicit
solvent. J. Chem. Phys..

[70] Batzner S., Musaelian A., Sun L., Geiger M., Mailoa J. P., Kornbluth M., Molinari N., Smidt T. E., Kozinsky B. (2022). E­(3)-equivariant
graph neural networks for data-efficient and accurate interatomic
potentials. Nat. Commun..

[71] Kim J., Kim H., Kim H., Lee D., Yoon S. (2025). A Comprehensive Survey
of Deep Learning for Time Series Forecasting: Architectural Diversity
and Open Challenges. Artif. Intell. Rev..

[72] Hénin J., Lelièvre T., Shirts M. R., Valsson O., Delemotte L. (2022). Enhanced sampling
methods for molecular dynamics simulations. Living J. Comput. Mol. Sci..

[73] Dietrich F. M., Advincula X. R., Gobbo G., Bellucci M. A., Salvalaglio M. (2024). Machine Learning
Nucleation Collective Variables with Graph Neural Networks. J. Chem. Theory Comput..

[74] Bonati L., Zhang Y.-Y., Parrinello M. (2019). Neural networks-based
variationally
enhanced sampling. Proc. Natl. Acad. Sci. U.
S. A..

[75] Rudzinski J. F. (2019). Recent
Progress towards Chemically-Specific Coarse-Grained Simulation Models
with Consistent Dynamical Properties. Computation.

[76] Wang Y., Voth G. A. (2025). Adversarial training
for dynamics matching in coarse-grained
models. J. Chem. Phys..

[77] Jin J., Schweizer K. S., Voth G. A. (2023). Understanding dynamics in coarse-grained
models. I. Universal excess entropy scaling relationship. J. Chem. Phys..

[78] Jin J., Schweizer K. S., Voth G. A. (2023). Understanding dynamics in coarse-grained
models. II. Coarse-grained diffusion modeled using hard sphere theory. J. Chem. Phys..

