# Supplementary material for: Probabilistic Forecasting for Coarse-Grained Molecular Dynamics
Source: J Chem Theory Comput. 2026 Apr 21;22(9):4231–46. doi: 10.1021/acs.jctc.5c02131 (PMC13157819; doi:10.1021/acs.jctc.5c02131)
Supplement: Supplementary file 1 [file ct5c02131_si_001.pdf]

Supplemental Information for:

Probabilistic Forecasting for Coarse-Grained Molecular Dynamics

Luc F. Christians[1], Anna Wojnar[1], Alexander J. Pak[1,2,3,*]

[1] Department of Chemical and Biological Engineering, Colorado School of Mines, Golden, CO, 80401, USA

[2] Quantitative Biosciences and Engineering Program, Colorado School of Mines, Golden, CO, 80401, USA

[3] Materials Science Program, Colorado School of Mines, Golden, CO, 80401, USA

[*] Corresponding author: apak@mines.edu

Table S1: Low-frequency all-atom molecular dynamics simulation parameters.

System	Chignolin	Trp-cage	A_5	A_{12}
**Box Length (nm)**	5.48	5.19	7.45	8.22
**Conc. NaCl (M)**	0.15	0.15	0.5	0.5
**Min. Tolerance (kJ/mol/nm)**	500	500	500	500
**T Damping Time (ps)**	2	2	2	2
**Sampling Rate (ps)**	5000	5000	100	100
**Replicas**	4	3	4	4
**Time Per Replica (ns)**	500	500	1,000	1,000
**Total Time (ns)**	2,000	1,500	4,000	4,000

Table S2: All-atom molecular dynamics equilibration parameters.

System	Chignolin	Trp-cage	A_5	A_{12}
**T equilibration time (ns)**	10	10	1	1
**NVT thermostat damping time (ps)**	0.1	0.1	0.1	0.1
**P equilibration time (ns)**	30	30	2	2
**NPT thermostat damping time (ps)**	0.5	0.5	0.2	0.2
**NPT barostat damping time (ps)**	5	5	2	2
**Temperature (K)**	420	270	300	300
**Pressure (bar)**	1	1	1	1

Table S3: High-frequency all-atom molecular dynamics simulation parameters.

System	Chignolin	Trp-cage	A_5	A_{12}
**T Damping Time (ps)**	2	2	2	2
**Sampling Rate (ps)**	0.05	0.05	0.05	0.05
**Replicas**	100	50	200	200
**Time Per Replica (ns)**	10	20	5	5
**Total Time (ns)**	1,000	1,000	1,000	1,000

Table S4: Local frame transformation reference CG sites (starting from index 0).

System	Chignolin	Trp-cage	A_5	A_{12}
**Reference 1**	3	12	3	5
**Reference 2**	0	18	1	1
**Reference 3**	7	4	4	7

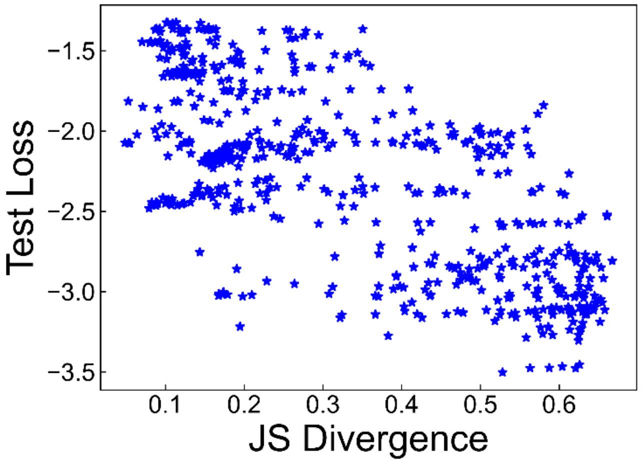

Figure S1: Comparing the Jensen-Shannon (JS) divergence to the test loss for converged Markovian PFCG polyalanine models.

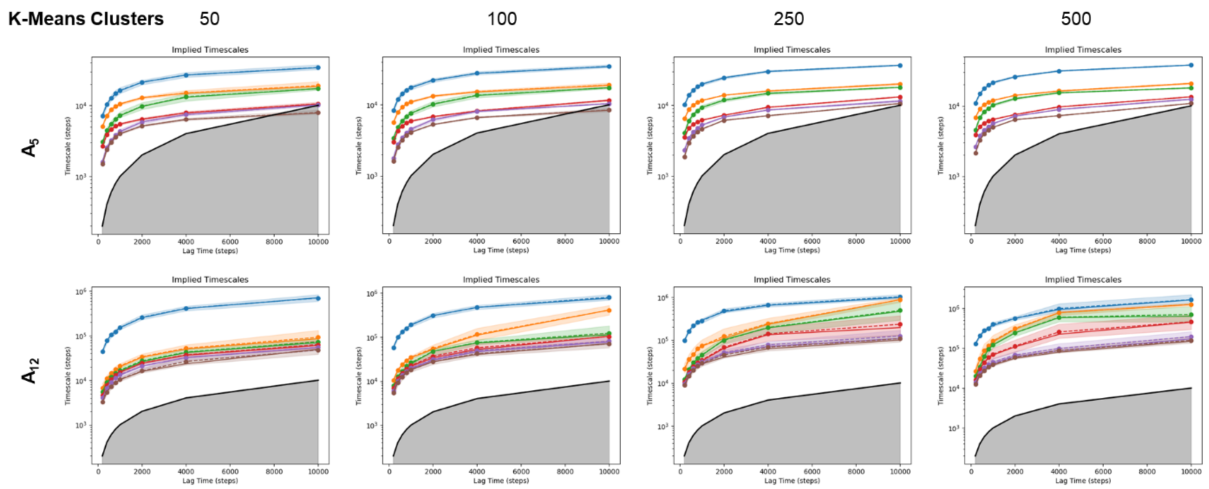

Figure S2: Implied time scale analysis for each polyalanine system for PCCA+ parameterization using different numbers of K-Means clusters as inputs over a range of MSM lag times.

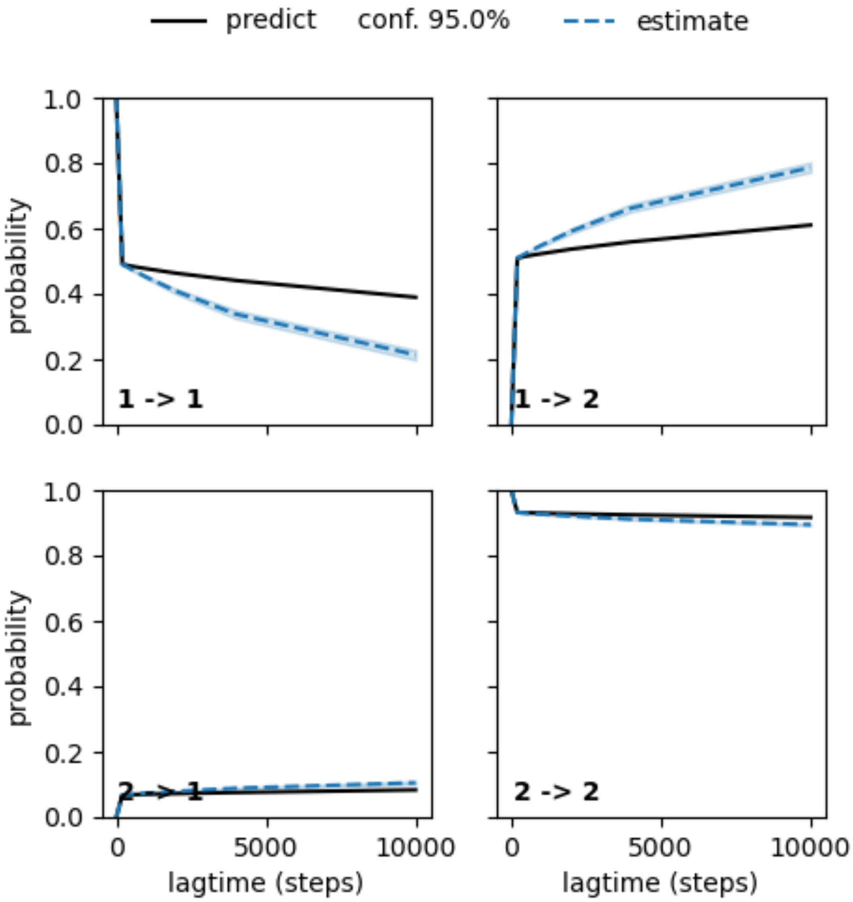

Figure S3: Chapman-Kolmogorov test profiles for the optimal A_5 model.

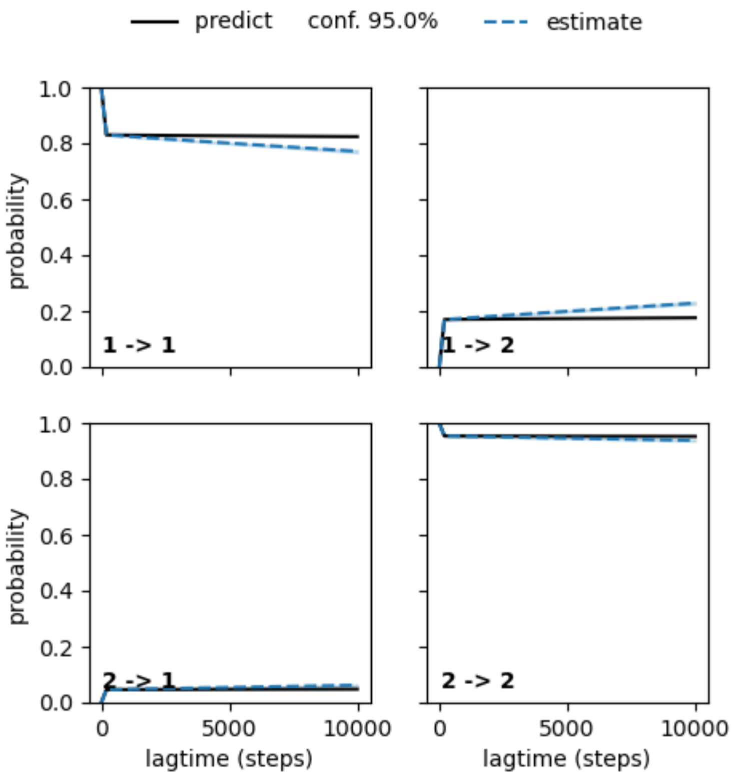

Figure S4: Chapman-Kolmogorov test profiles for the optimal A_{12} model.

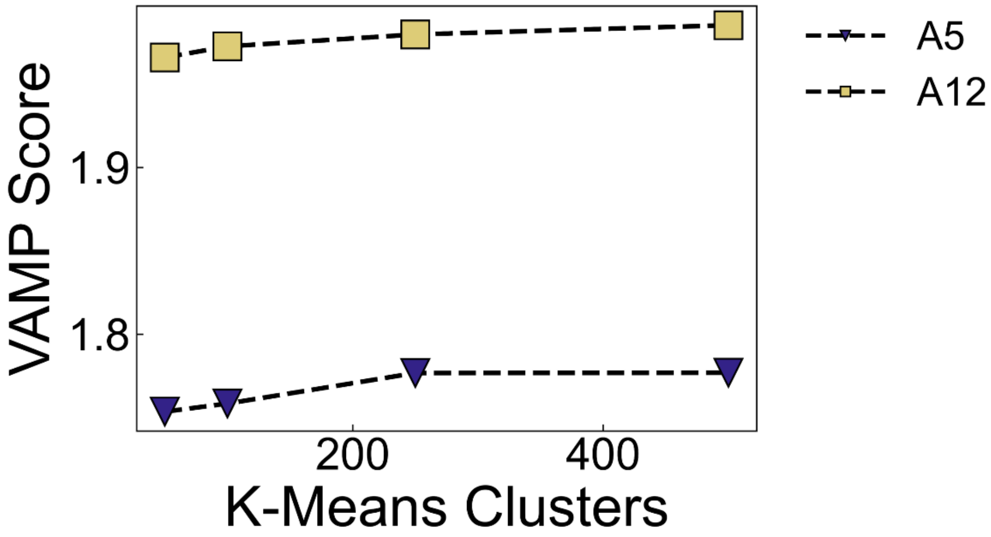

Figure S5: VAMP scores for MSMs at a lag time of 10 ps (2,000 steps) for each polyalanine system.

Table S5: PFCG optimized model hyperparameters.

System	Chignolin	Trp-cage	A_5	A_{12}
**Gaussian noise**	0.01	0.02	0.01	0.01
**Time steps (ps)**	1.0, 2.5, 10.0	1.0, 2.5, 10.0	1.0	10.0
**Markovian block attention heads**	5	4	2	2
**Markovian block dimensions**	64	128	64	32
**Markovian block hidden dimensions**	256	256	256	256
**Markovian block layers**	3	2	1	2
**Markovian block dropout rate**	0.2	0.2	0.2	0.2
**non-Markovian block attention heads**	5	4	2	2
**non-Markovian block dimensions**	64	128	64	32
**non-Markovian block hidden dimensions**	256	256	256	256
**non-Markovian block layers**	3	2	1	2
**non-Markovian block dropout rate**	0.2	0.2	0.2	0.2
**Hidden State dimensions**	90	120	30	144
**Tested non-Markovian Sequence Lengths**	5, 15, 40, 75	5, 15, 40, 75	5,10, 40, 100	5, 10, 40, 100

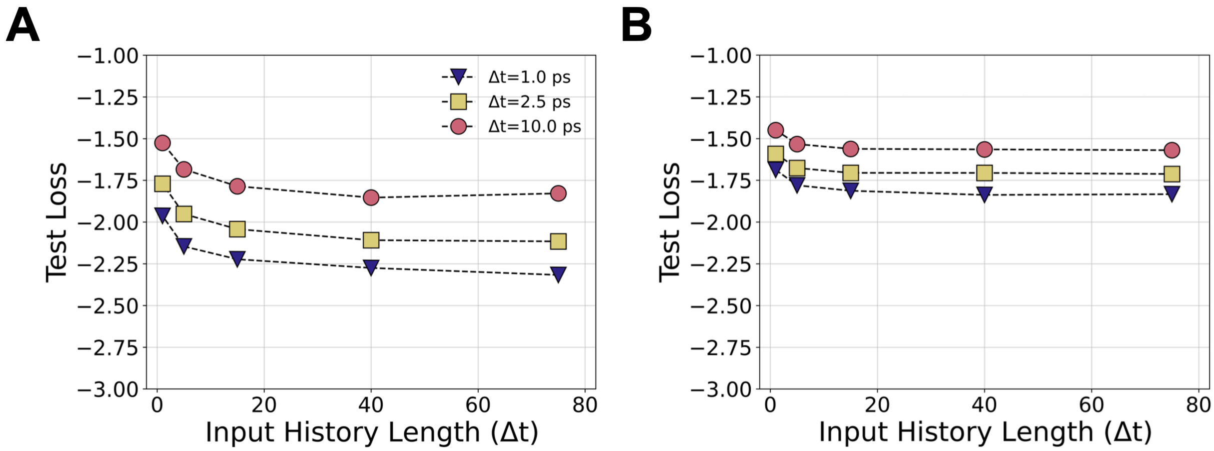

Figure S6: Test loss as a function of input history length over tested timesteps for (A) chignolin and (B) Trp-cage.

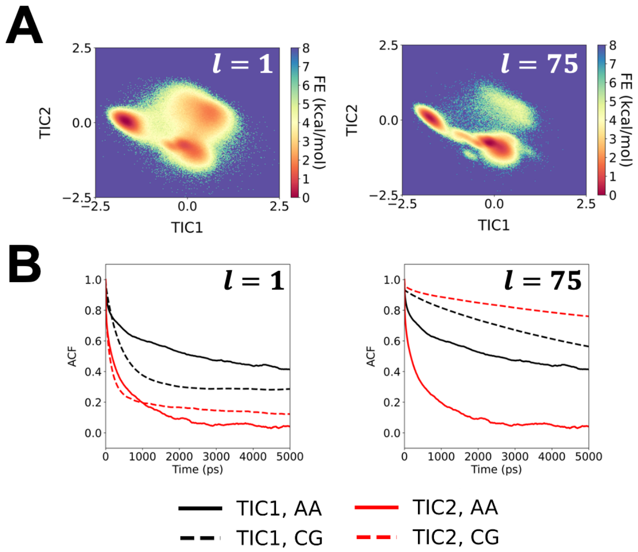

Figure S7: (A) 2D FESs and (B) ACF curves generated from PFCG trajectories of chignolin using Δt = 0.05 ps, with input history length $l = 1$ (left) and $l = 75$ (right).

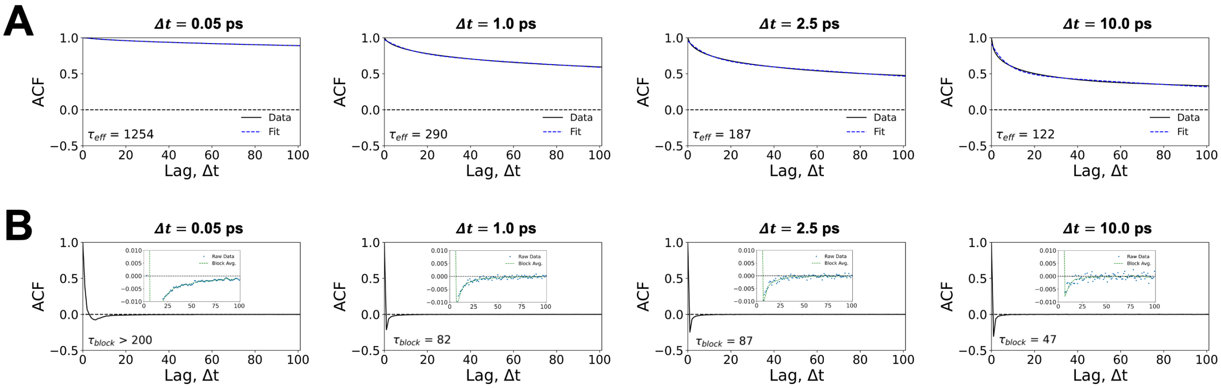

Figure S8: (A) ACF profiles for chignolin averaged across all unscaled LFT features as a function of lag in number of timesteps, with timestep ranging from Δt = 0.05 ps (left) to Δt = 10.0 ps (right). The profiles are fit to a double exponential, i.e., $A_1 \exp(-t/\tau_1) + A_2 \exp(-t/\tau_2)$, and $\tau_{eff} = (A_1\tau_1 + A_2\tau_2)/(A_1 + A_2)$. (B) ACF profiles for chignolin averaged across all time-differenced unscaled LFT features, where we show the decorrelation to zero in the inset. The decorrelation time (τ_{block}) is calculated as the lag time of the block-averaged ACF profile where convergence to zero within a tolerance of 5×10^{-5} is observed, using a block size of 5.

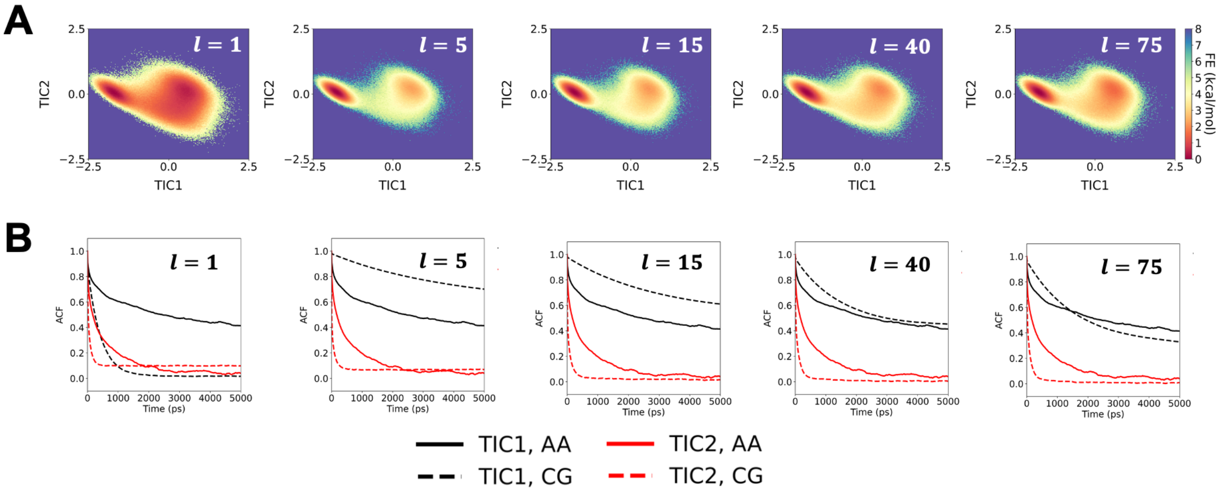

Figure S9: (A) 2D FESs and (B) ACF curves generated from PFCG trajectories of chignolin using Δt = 1.0 ps, with input history length ranging from $l = 1$ (left) to $l = 75$ (right).

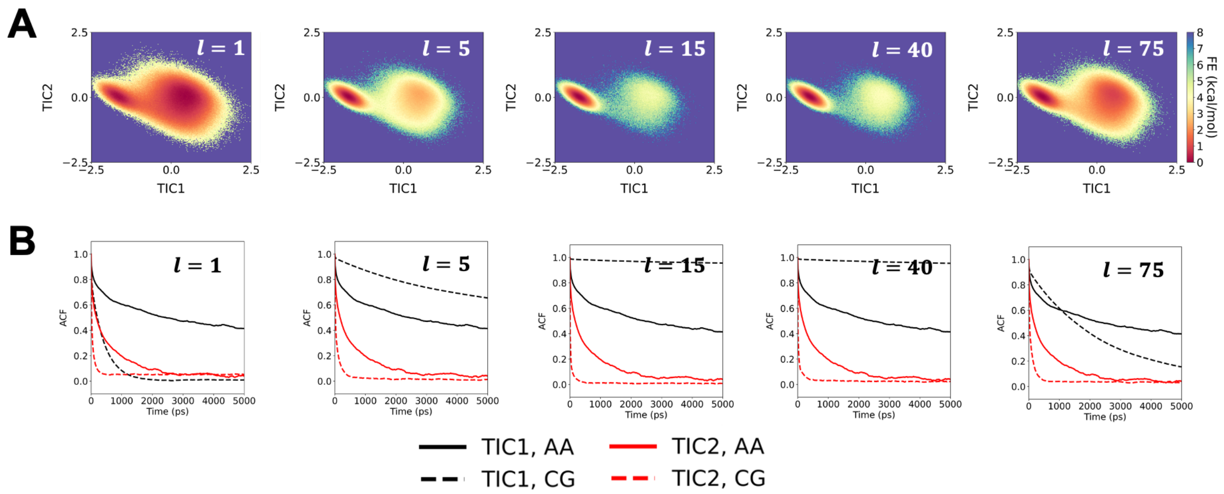

Figure S10: (A) 2D FESs and (B) ACF curves generated from PFCG trajectories of chignolin using Δt = 2.5 ps, with input history length ranging from $l = 1$ (left) to $l = 75$ (right).

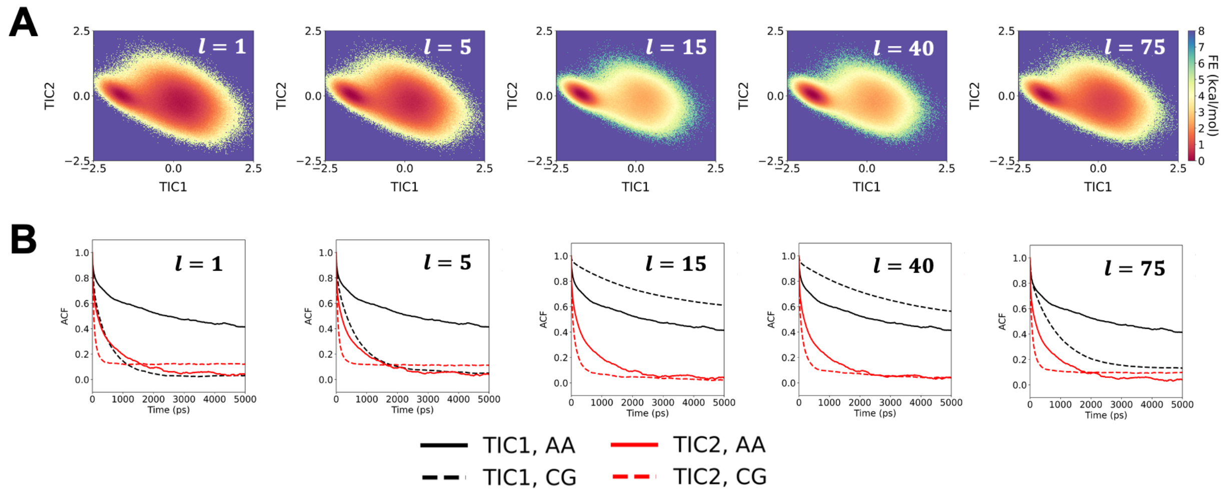

Figure S11: (A) 2D FESs and (B) ACF curves generated from PFCG trajectories of chignolin using Δt = 10.0 ps, with input history length ranging from $l = 1$ (left) to $l = 75$ (right).

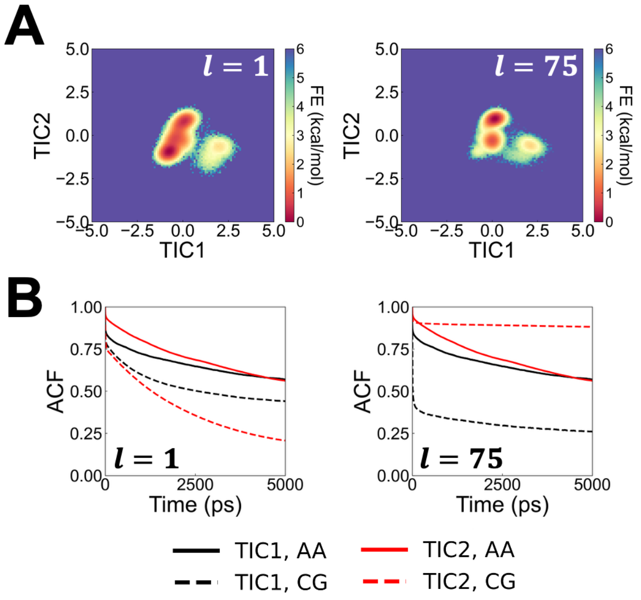

Figure S12: (A) 2D FESs and (B) ACF curves generated from PFCG trajectories of Trp-cage using Δt = 0.05 ps, with input history length $l = 1$ (left) and $l = 75$ (right).

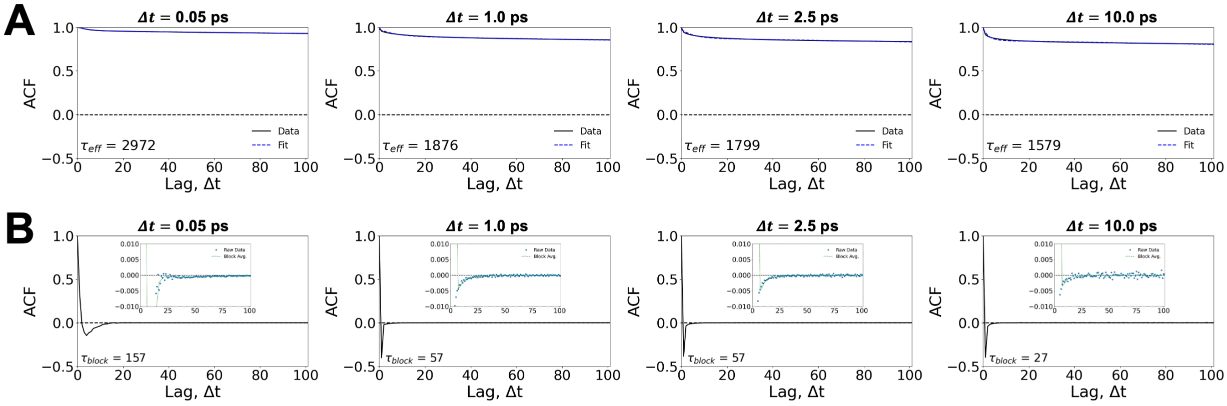

Figure S13: (A) ACF profiles for Trp-cage averaged across all unscaled LFT features as a function of lag in number of timesteps, with timestep ranging from Δt = 0.05 ps (left) to Δt = 10.0 ps (right). The profiles are fit to a double exponential, i.e., $A_1 \exp(-t/\tau_1) + A_2 \exp(-t/\tau_2)$, and $\tau_{eff} = (A_1\tau_1 + A_2\tau_2)/(A_1 + A_2)$. (B) ACF profiles for Trp-cage averaged across all time-differenced unscaled LFT features, where we show the decorrelation to zero in the inset. The decorrelation time (τ_{block}) is calculated as the lag time of the block-averaged ACF profile where convergence to zero within a tolerance of 5×10^{-5} is observed, using a block size of 5.

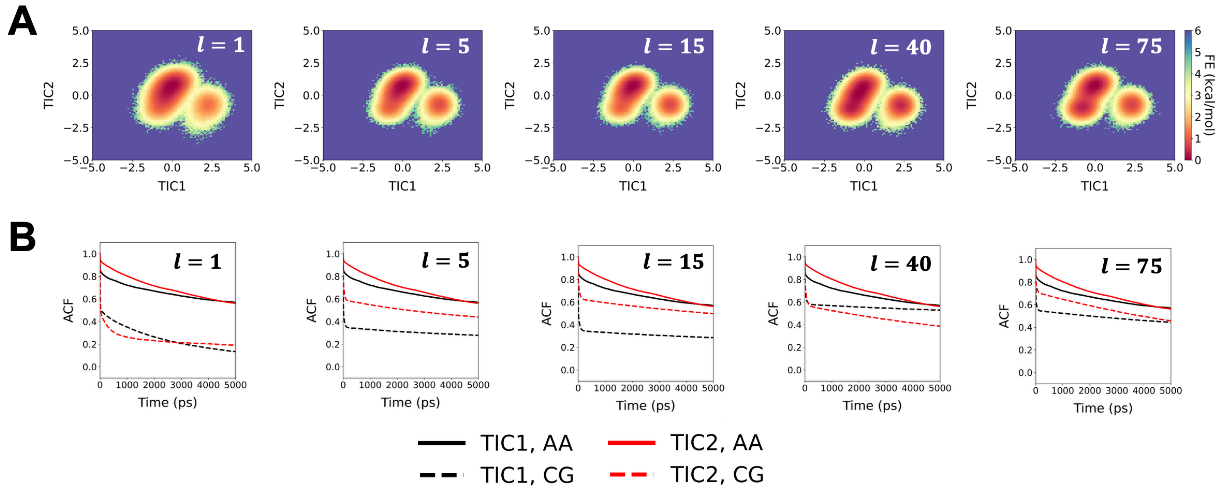

Figure S14: (A) 2D FESs and (B) ACF curves generated from PFCG trajectories of Trp-cage using Δt = 1.0 ps, with input history length ranging from $l = 1$ (left) to $l = 75$ (right).

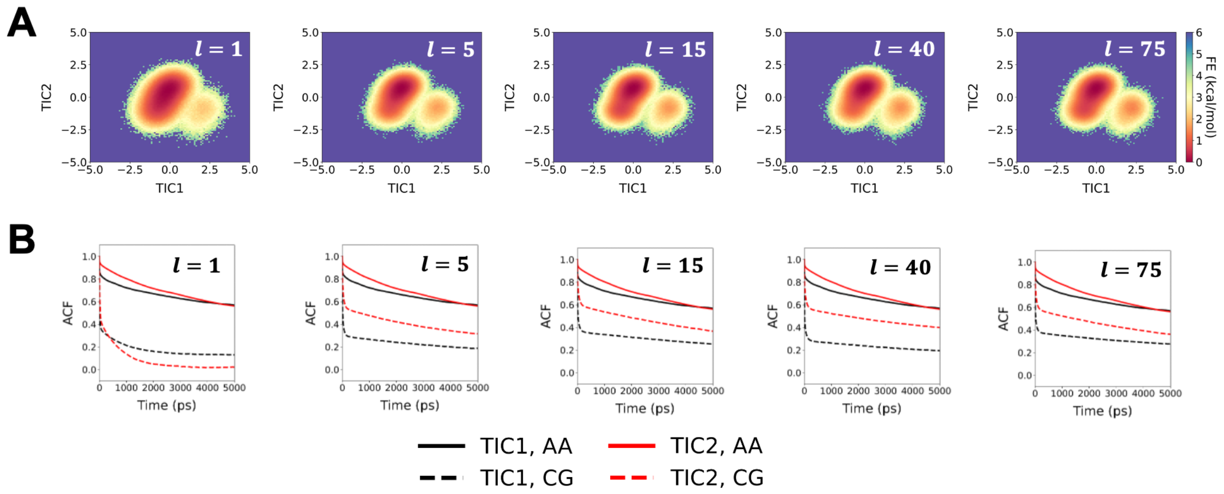

Figure S15: (A) 2D FESs and (B) ACF curves generated from PFCG trajectories of Trp-cage using Δt = 2.5 ps, with input history length ranging from $l = 1$ (left) to $l = 75$ (right).

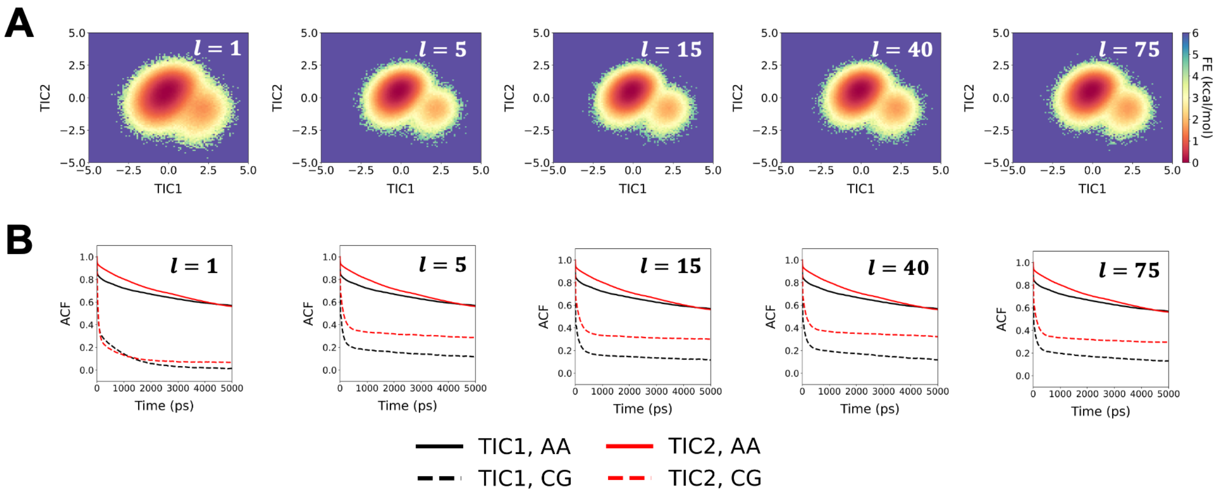

Figure S16: (A) 2D FESs and (B) ACF curves generated from PFCG trajectories of Trp-cage using Δt = 10.0 ps, with input history length ranging from $l = 1$ (left) to $l = 75$ (right).

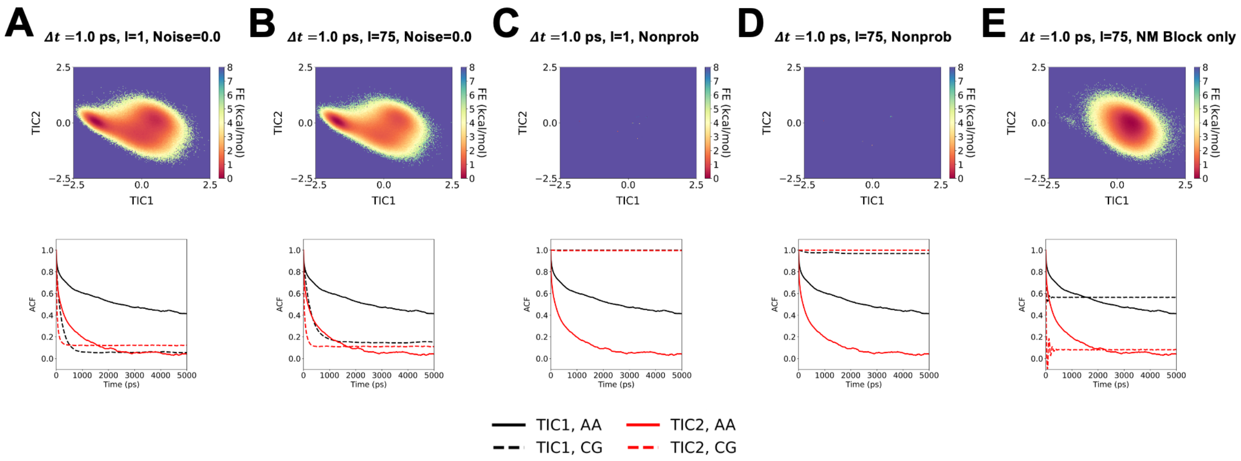

Figure S17: (A-E) Ablation study figures for chignolin at Δt = 1.0 ps with the FESs (top) and ACF curves (bottom) shown for each listed ablation study condition and model type.

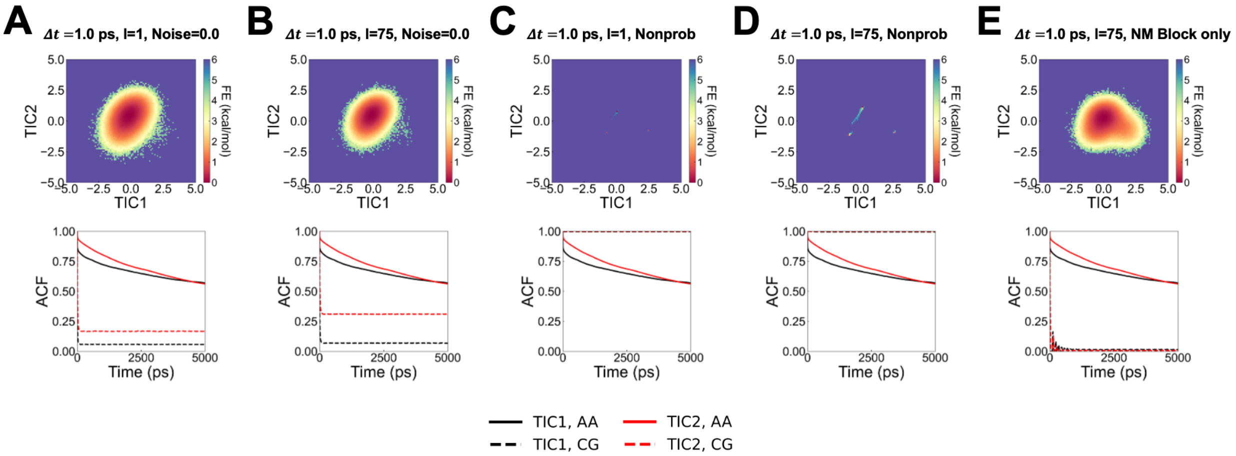

Figure S18: (A-E) Ablation study figures for Trp-cage at Δt = 1.0 ps with the FESs (top) and ACF curves (bottom) shown for each listed ablation study condition and model type.

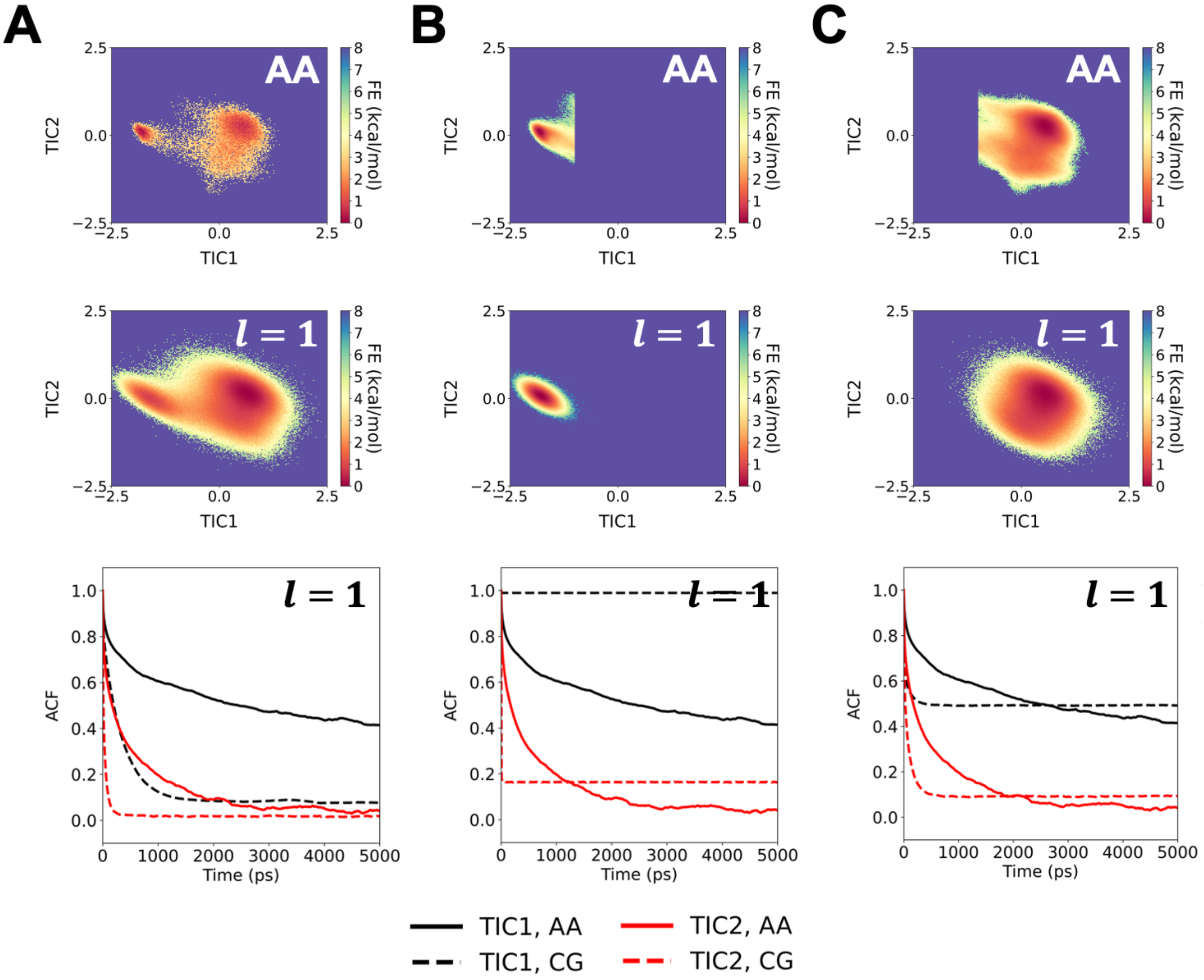

Figure S19: Chignolin PFCG results under limited data conditions at Δt = 1.0 ps with l = 1. (A-C) Comparison of training data FESs (top), PFCG generated FESs (middle), and comparison of ACF curves for TIC1 and TIC2 calculated from AA MD and PFCG (bottom) under conditions of (A) sparse training data, (B) training data where only the folded state is sampled, and (C) training data where only the unfolded state is sampled.

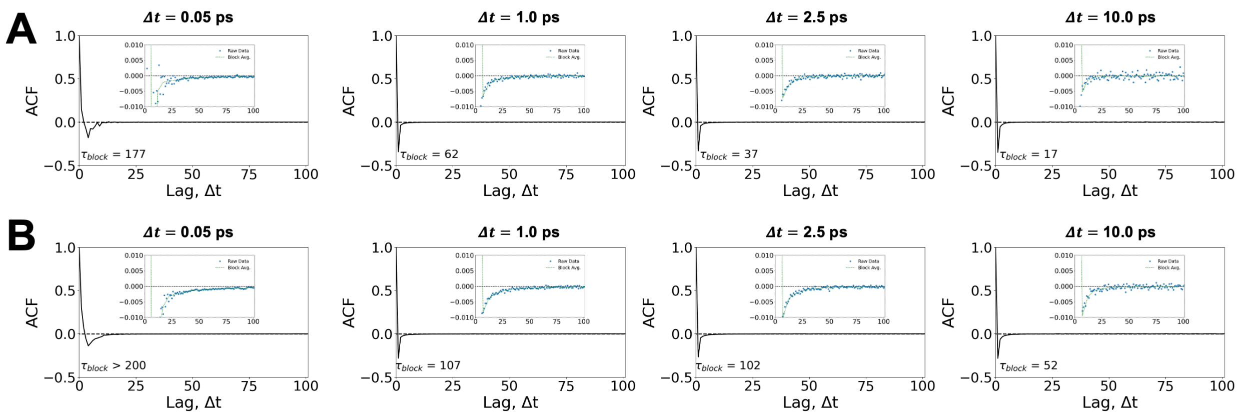

Figure S20: (A) A_5 and (B) A_{12} ACF profiles averaged across all time-differenced unscaled LFT features, where we show the decorrelation to zero in the inset. The decorrelation time (τ_{block}) is calculated as the lag time of the block-averaged ACF profile where convergence to zero within a tolerance of 5×10^{-5} is observed, using a block size of 5.

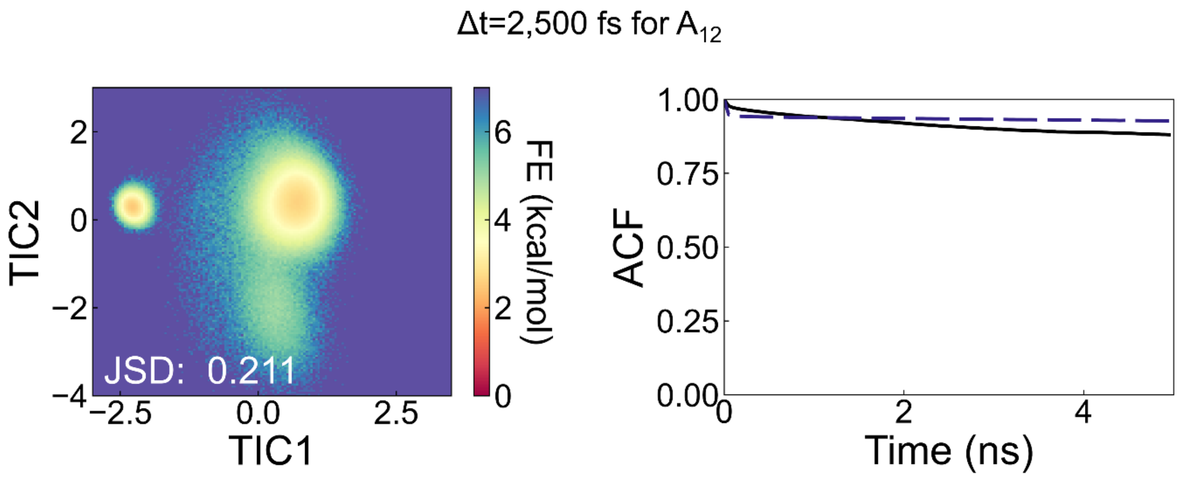

Figure S21: A_{12} Markovian PFCG results for the optimized architecture using a timestep of 2,500 fs.

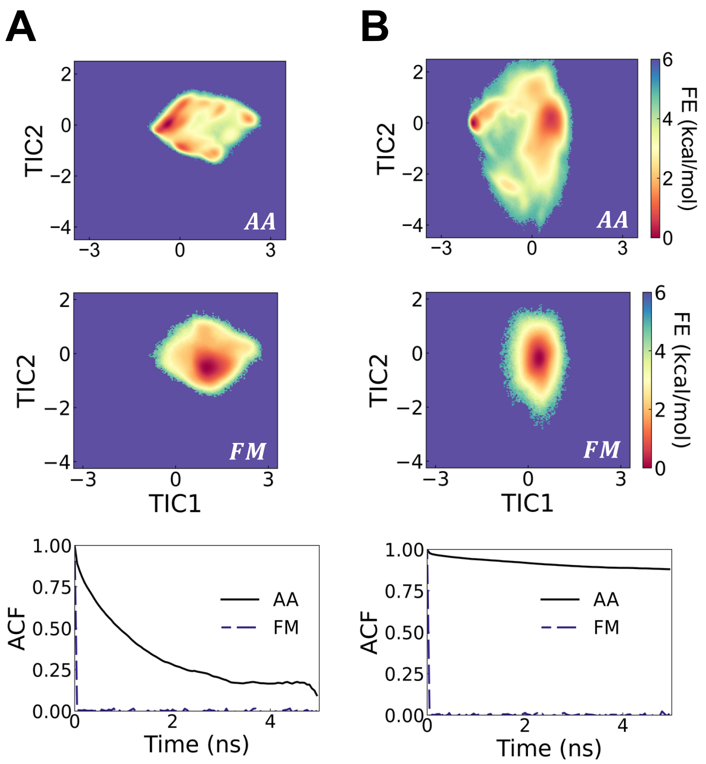

Figure S22: Simulation data for a traditional two-body (pairwise) interaction model trained using the force matching approach, as compared to atomistic simulation statistics using free energy (FE) surfaces (upper) and autocorrelation function (ACF) curves (lower) for (A) A_5 and (B) A_{12}.

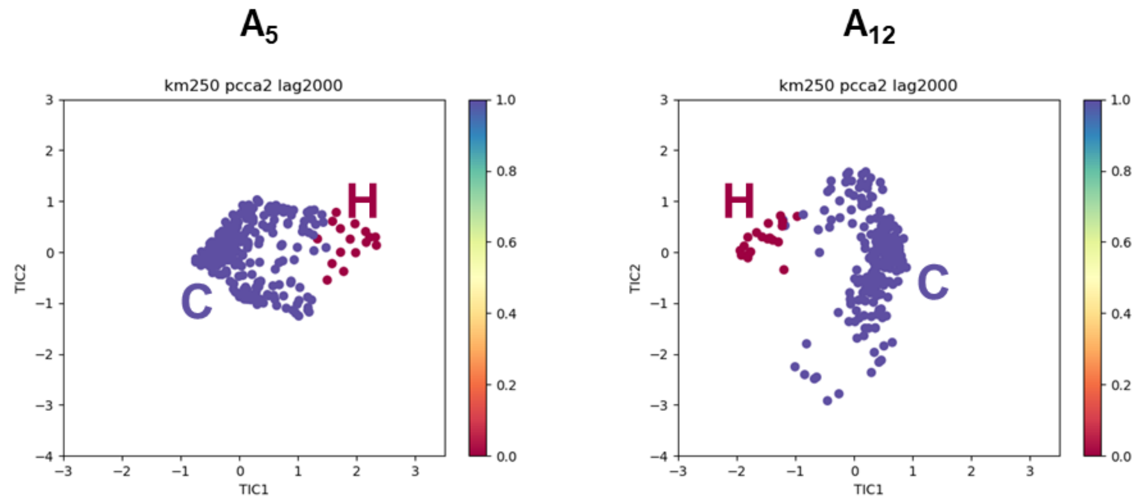

Figure S23: K-Means clusters colored by their PCCA+ metastate for the optimized models of each listed polyalanine system. The 'H' state represents the helical state and the 'C' state represents the random-coil state.

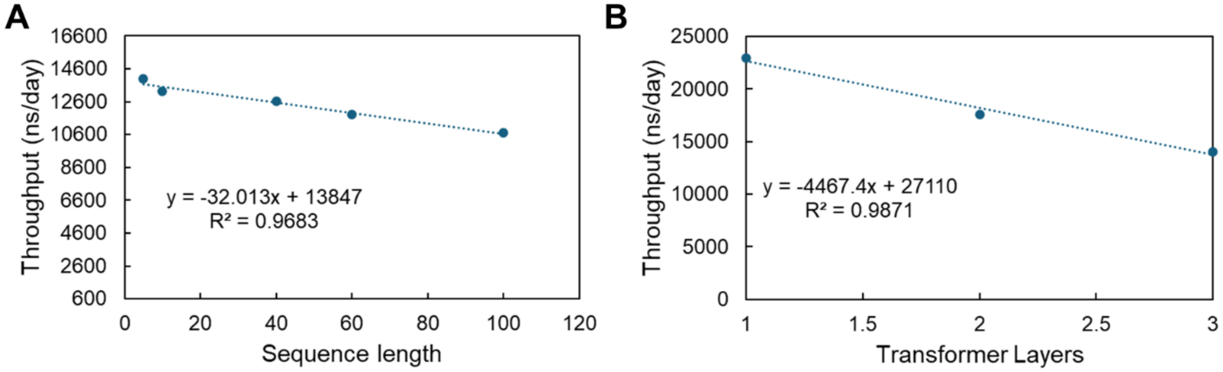

Figure S24: The impact of varying (A) the sequence length for the non-Markovian models and (B) the number of layers for the Markovian models on inference throughput in ns/day for A_{12}.
